# Supplementary material for: Single-cell transcriptomics identifies CD44 as a marker and regulator of endothelial to haematopoietic transition
Source: Nat Commun. 2020 Jan 29;11:586. doi: 10.1038/s41467-019-14171-5 (PMC6989687; doi:10.1038/s41467-019-14171-5)
Supplement: Supplementary file 1 — Supplementary Information [file 41467_2019_14171_MOESM1_ESM.pdf]

Supplementary Information for:

Single-cell transcriptomics identifies CD44 as a new marker and regulator of endothelial to haematopoietic transition

Oatley *et al.*

Supplementary Figure 1

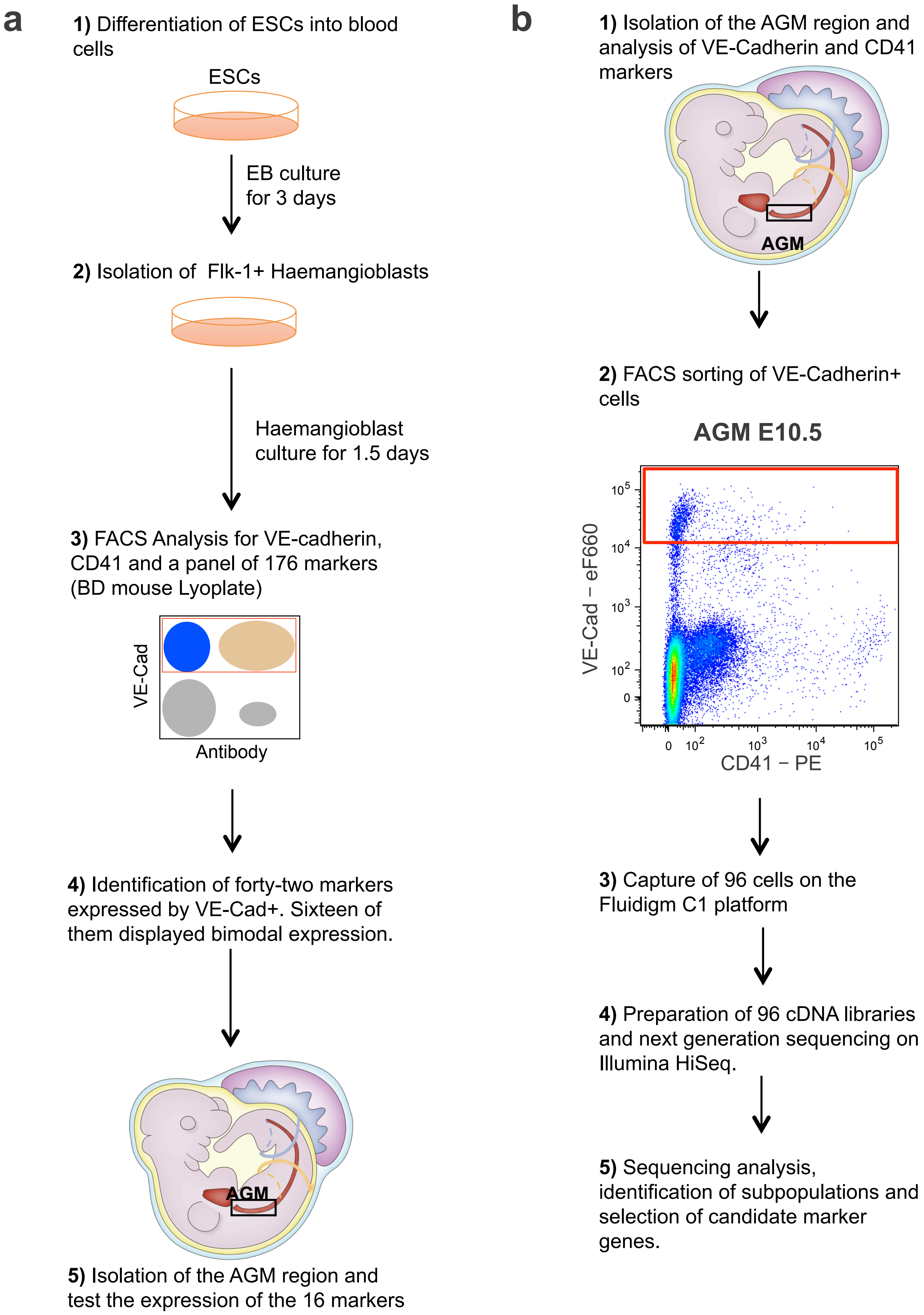

Supplementary Figure 1: Experimental layout for the experiments for antibody screen and single-cell RNA sequencing. (**a**) Strategy used for the antibody screen. (**b**) Description of the different steps following for the single cell RNA sequencing analysis in the AGM. See also Figure 1 and Supplementary Table 1.

Supplementary Figure 2

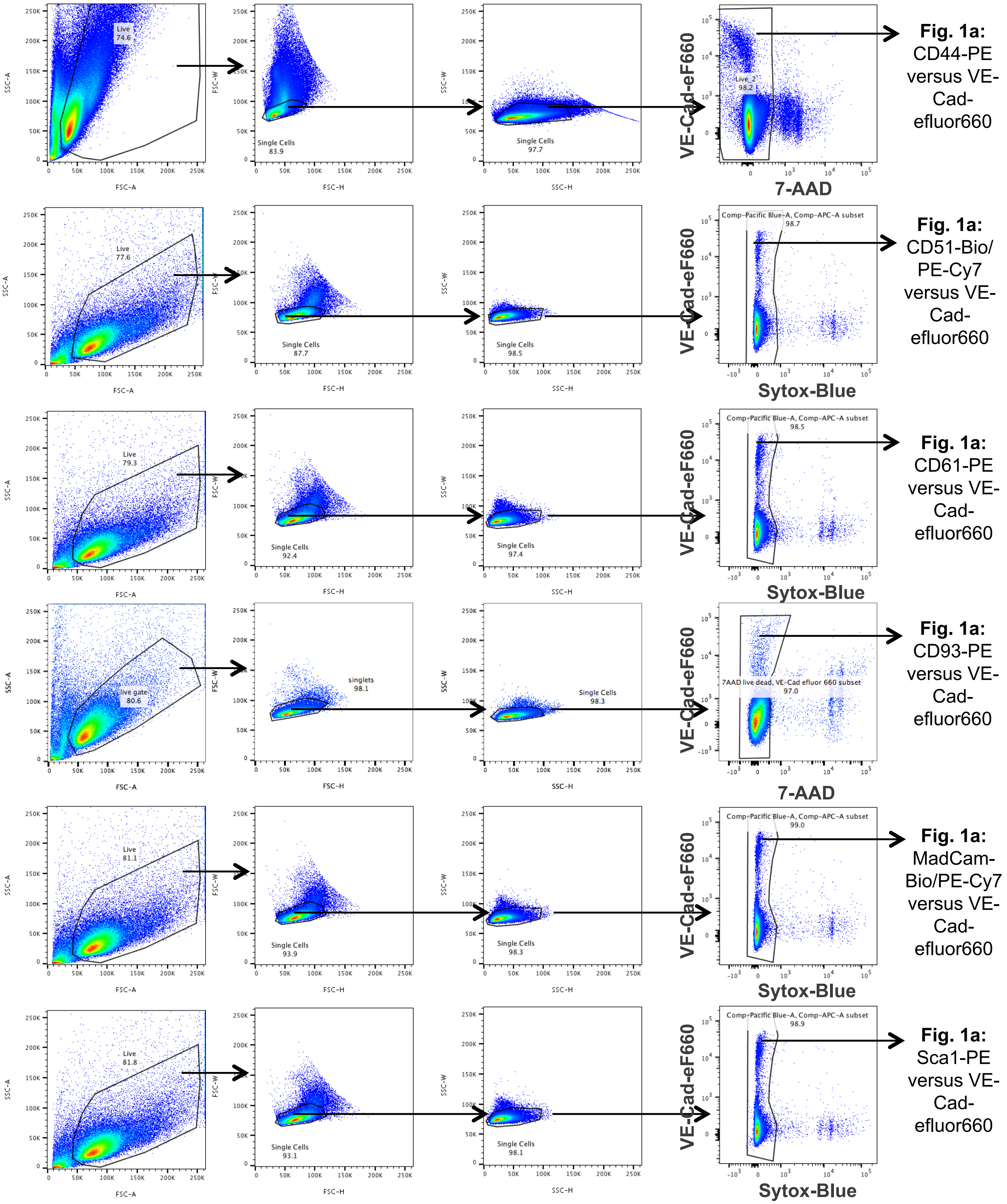

Supplementary Figure 2: Gating strategies using for FACS analysis shown in Figure 1a.
From left to right: FSC-A versus SSC-A, FSC-H versus FSC-W, SSC-H versus SSC-W and VE-Cad-eF660 versus dead cell dye 7-AAD or Sytox Blue. The arrows indicate the cells selected from one plot to the next.

Supplementary Figure 3

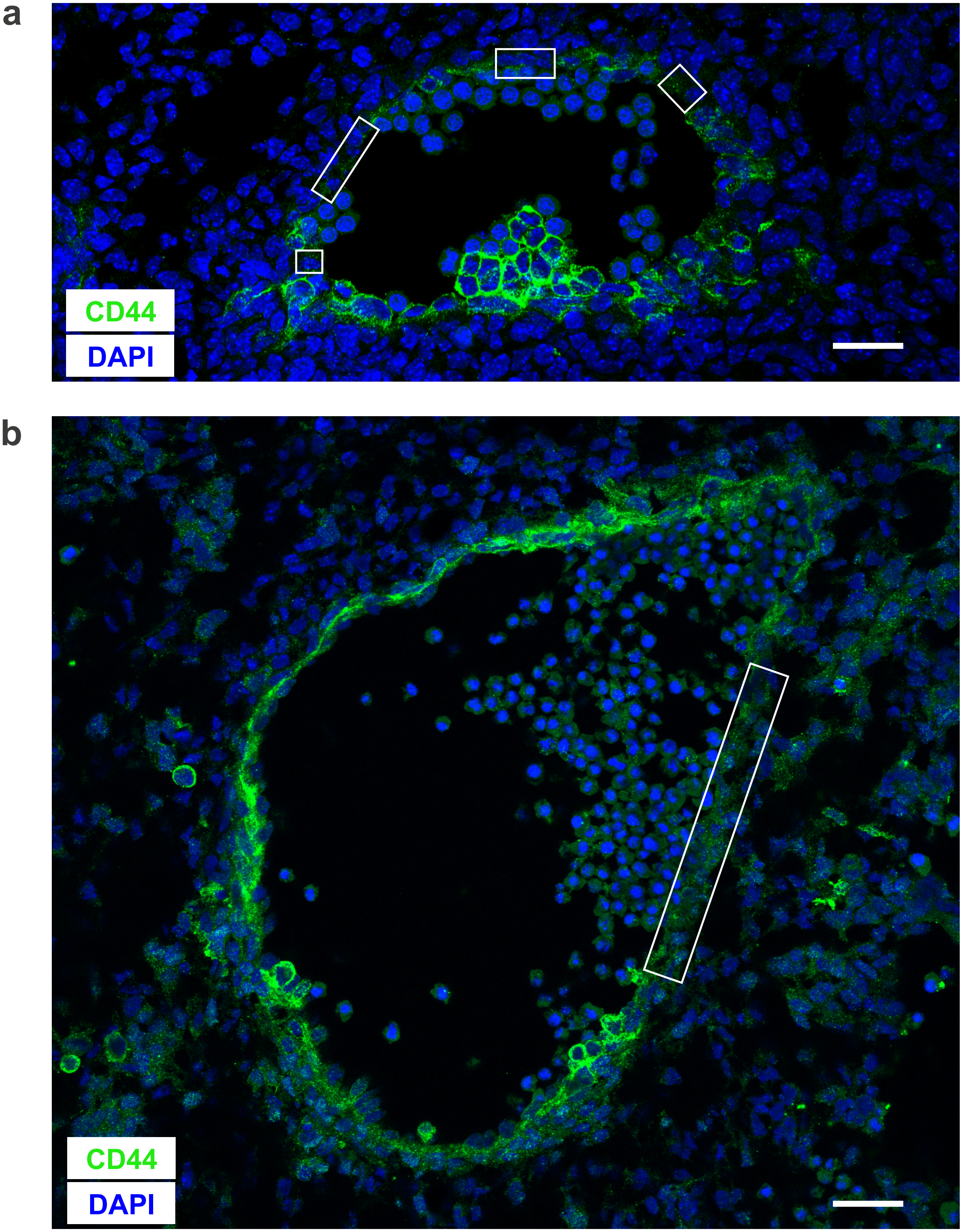

Supplementary Figure 3: Immunofluorescence analysis of CD44 in the AGM region at E10 and E11.
(**a**) CD44 expression in the dorsal aorta at E10. Boxes indicate CD44 Negative areas. (**b**) CD44 expression in the dorsal aorta at E11. Boxes indicate CD44 Negative areas. Scale bars correspond to 30 μm. See also Figure 2a.

Supplementary Figure 4

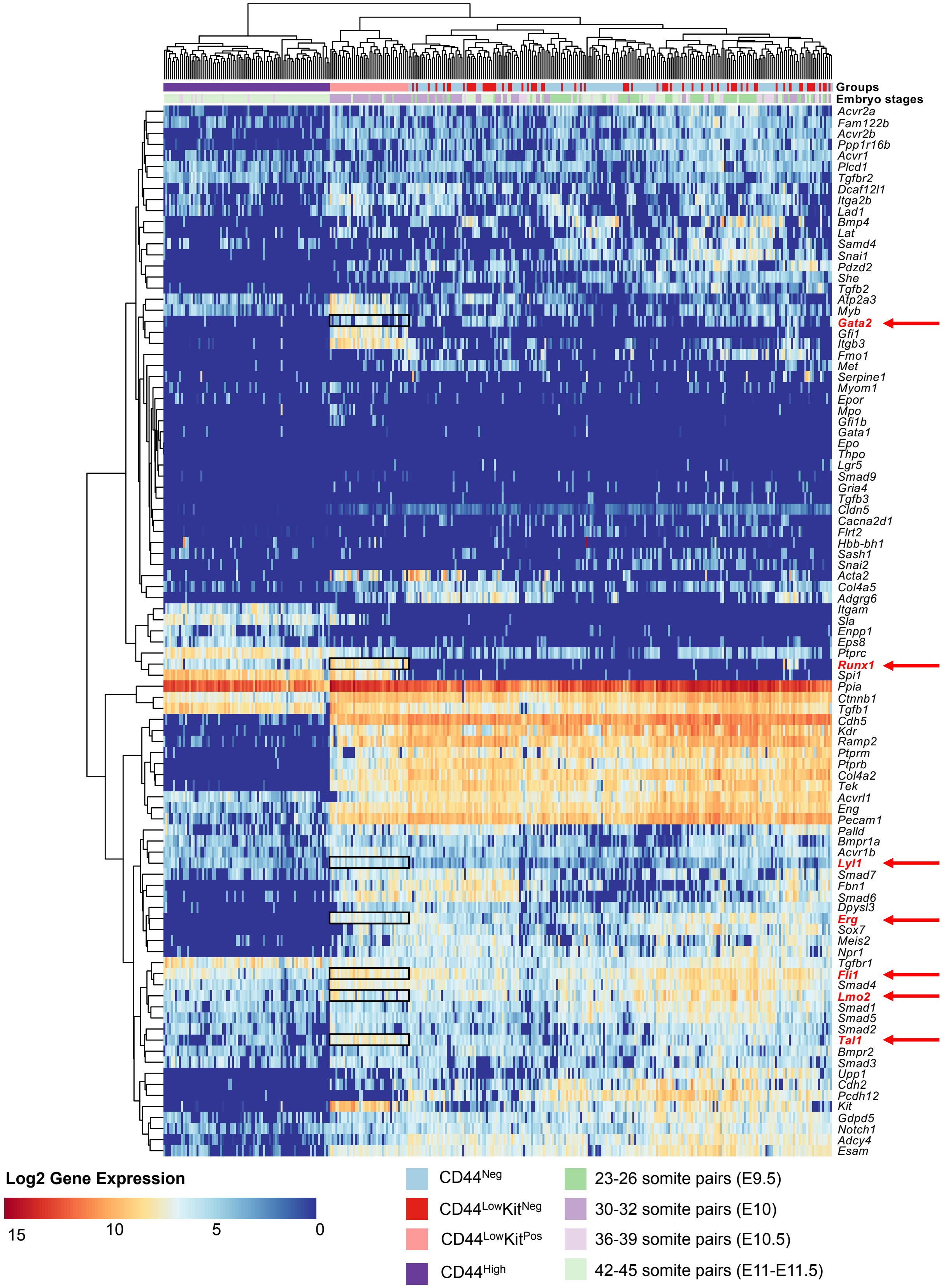

Supplementary Figure 4: Single-cell q-RT-PCR analysis of the four populations defined by CD44, VE-Cad and Kit. Single cells from indicated populations were isolated and tested for the expression of 95 genes by single-cell q-RT-PCR. The heatmap shows the result of the hierarchical clustering analysis (cells were clustered by Euclidian distance and the genes by Pearson correlation). Genes coding for *Gata2*, *Runx1*, *Lyl1*, *Lmo2*, *Tal1*, *Fli1* and *Erg* transcription factors are specifically co-expressed in the CD44^{Low}Kit^{Pos} population but not in the other two (see genes indicated by red arrows). See also Figure 3 and Supplementary Data 2.

Supplementary Figure 5

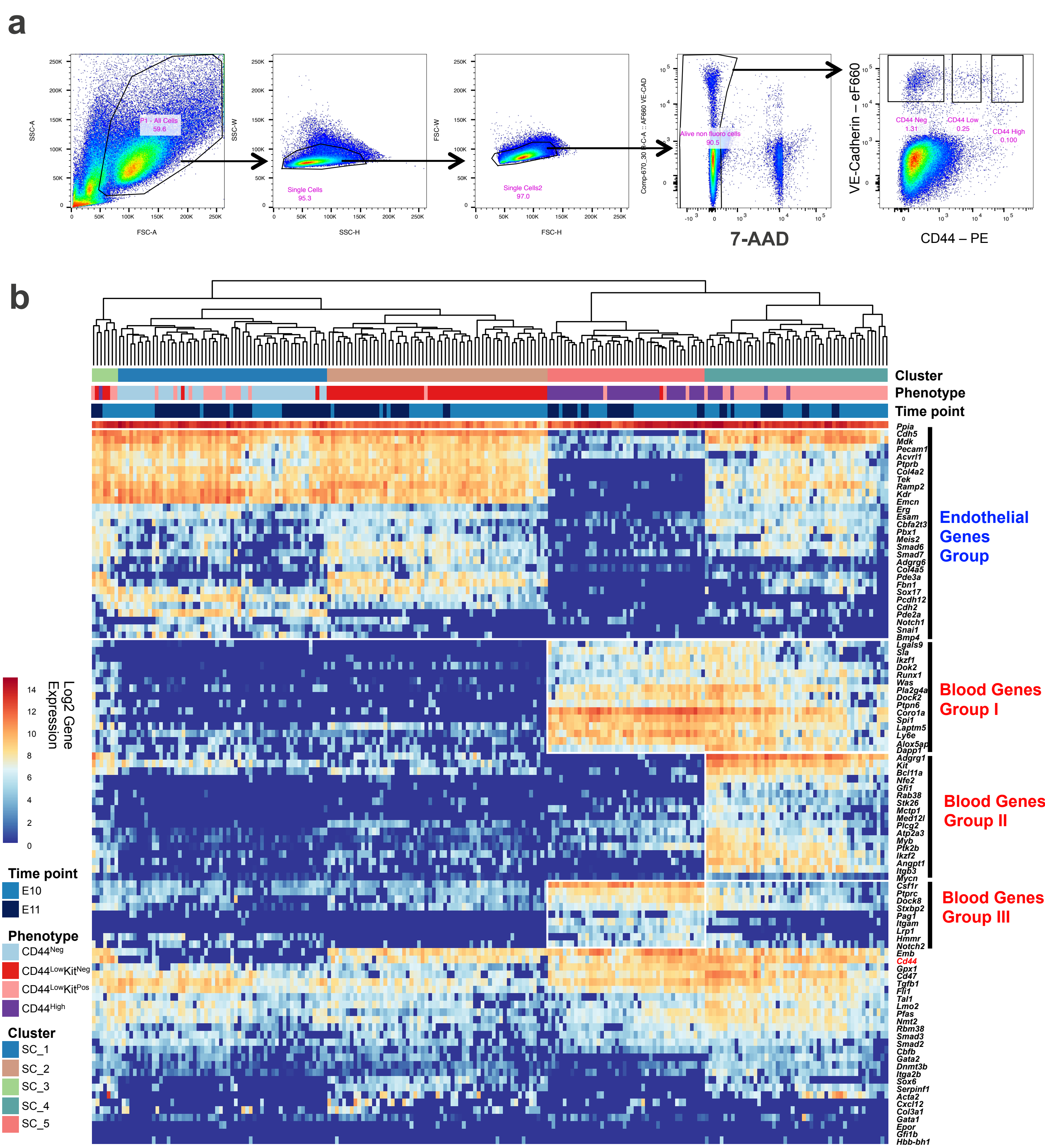

Supplementary Figure 5: Expression of VE-Cad, CD44 and Kit defines transcriptionally distinct cell populations.

(**a**) FACS gating strategy for the CD44 populations shown in Fig. 2. (**b**) Single cells from each VE-Cad+ population were isolated and tested for the expression of 96 genes by single-cell q-RT-PCR. The heatmap shows the result of the hierarchical clustering analysis (cells were clustered by Euclidian distance). A selection of genes has been highlighted: endothelial genes group, blood genes groups I, II and III. See also Figure 3 and Supplementary Data 3.

Supplementary Figure 6

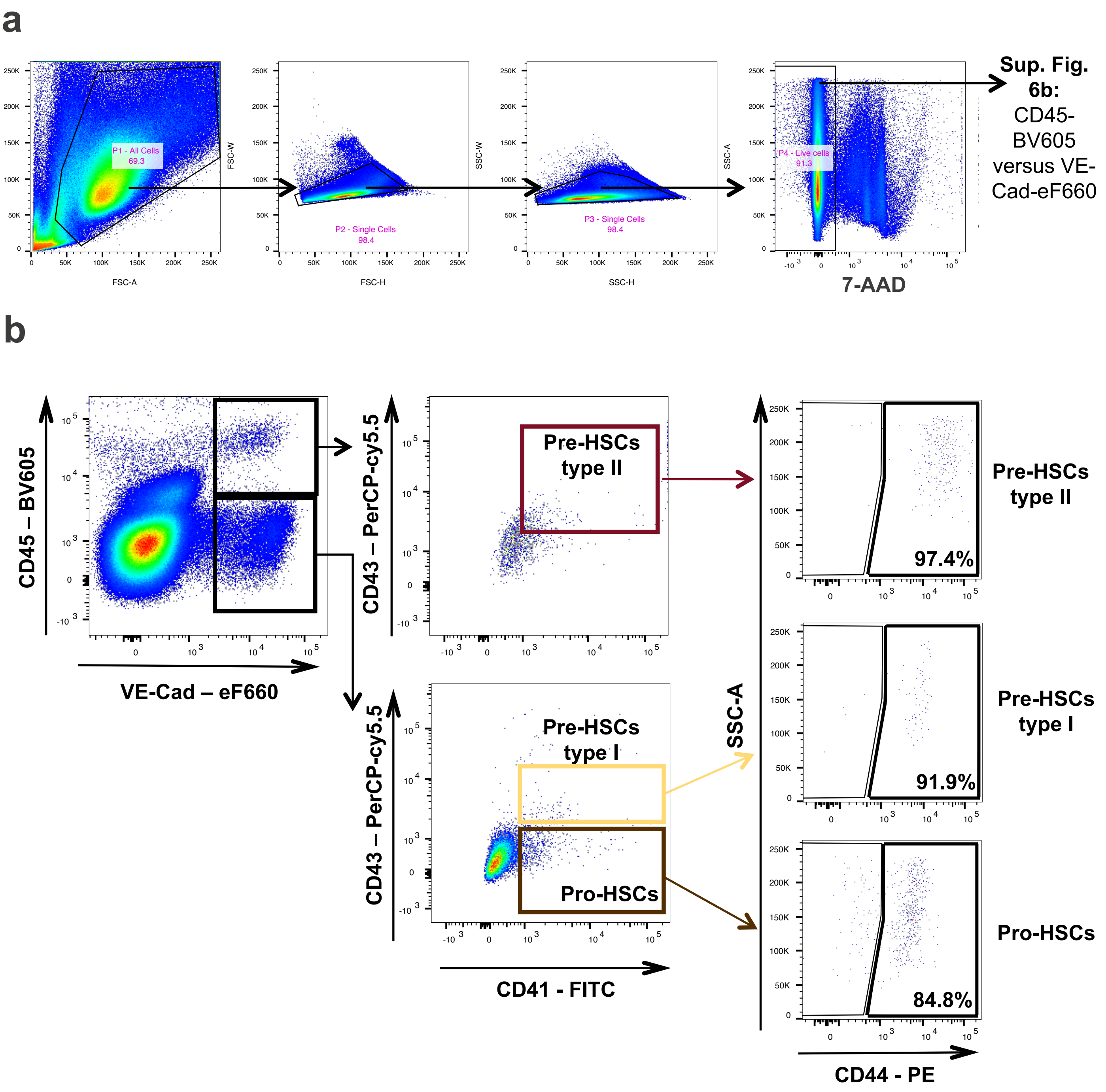

Supplementary Figure 6: Pro-HSC, Pre-HSC-I and Pre-HSC-II are expressing CD44 protein.
(**a**) FACS gating strategy for pro-HSC, pre-HSC type I and pre-HSC type II. (**b**) Pro-HSCs (VE-Cad$^+$ CD41$^+$ CD45$^-$ CD43$^-$), Pre-HSC-I (VE-Cad$^+$ CD41$^+$ CD45$^-$ CD43$^+$), Pre-HSC-II (VE-Cad$^+$ CD45$^+$) populations were analysed by FACS analysis to assess the expression of CD44 protein. See also Figure 4b.

Supplementary Figure 7

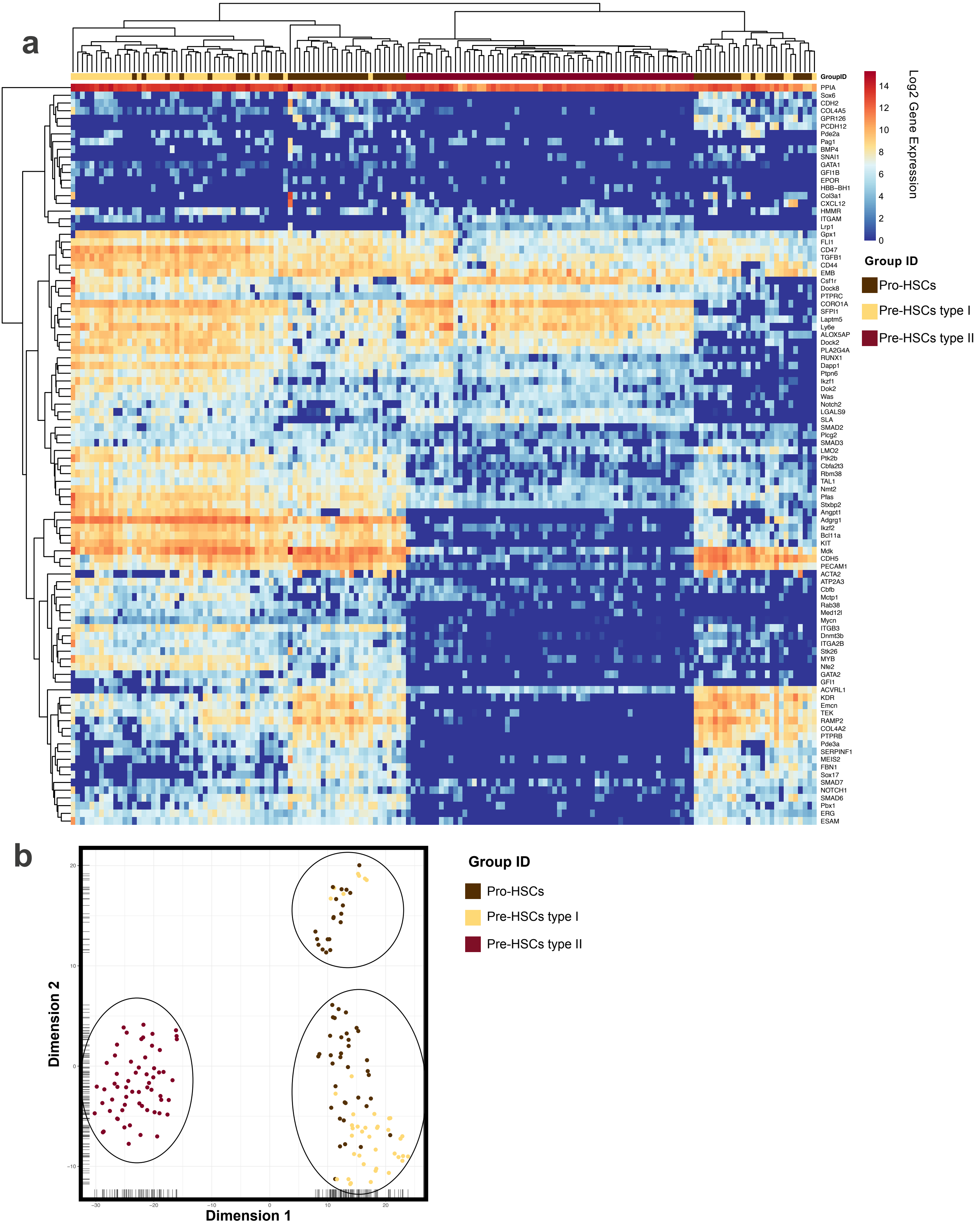

Supplementary Figure 7: Results of single cell q-RT-PCR analysis of Pro-HSC, Pre-HSC-I and Pre-HSC-II.
(**a**) Single cells from Pro-HSCs (VE-Cad$^+$ CD41$^+$ CD45$^-$CD43$^-$), Pre-HSC-I (VE-Cad$^+$ CD41$^+$ CD45$^-$ CD43$^+$), Pre-HSC-II (VE-Cad$^+$ CD45$^+$) populations were isolated and tested by single-cell q-RT-PCR. The heatmap shows the result of the hierarchical clustering analysis (cells were clustered by Euclidian distance and the genes by Pearson correlation). (**b**) tSNE plot from single cell q-RT-PCR data shown in (a). See also Figure 4 and Supplementary Data 4.

Supplementary Figure 8

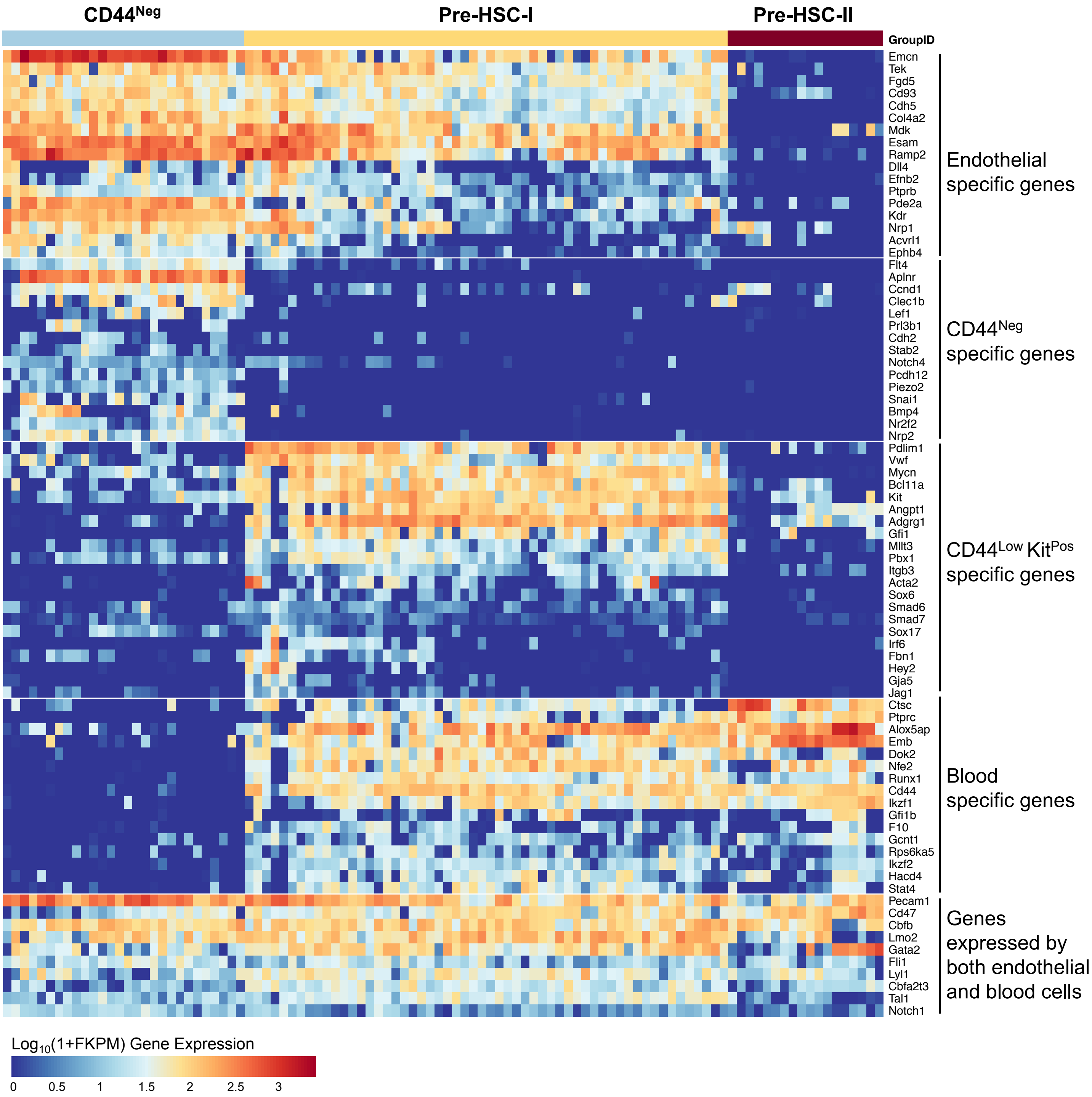

Supplementary Figure 8: Analysis of the Zhou et al. sc-RNA-seq dataset.
The heatmap shows the expression pattern of genes selected from Fig. 3 and Fig. 5 in the single cells studied by Zhou et al. [14] using sc-RNA-seq. These cells were isolated from E11 mouse embryos and included endothelial cells (CD44 Neg), Pre-HSC-I and Pre-HSC-II. The genes were grouped in the five indicated categories. See also Supplementary Data 6.

Supplementary Figure 9

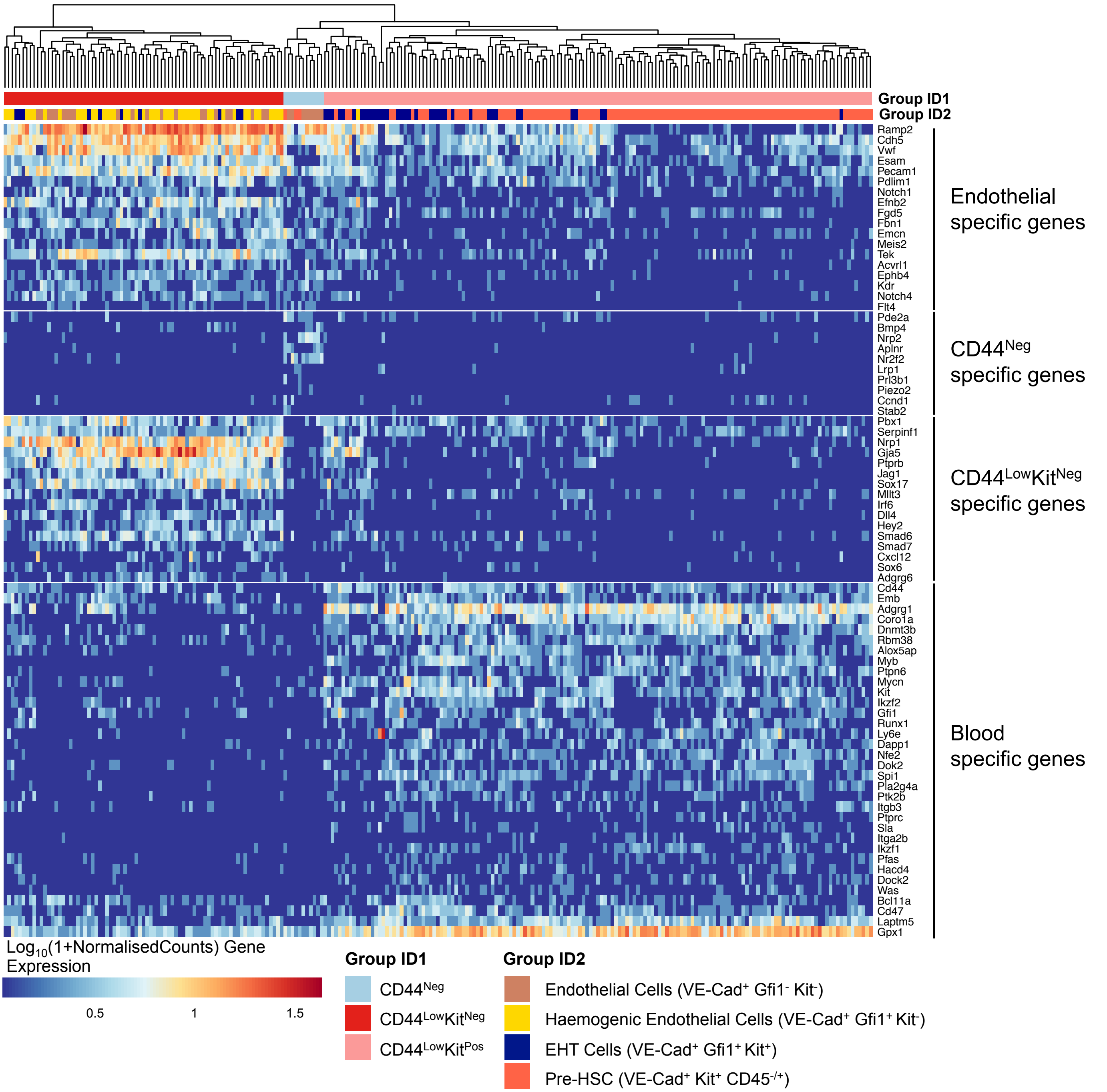

Supplementary Figure 9: Analysis of the Baron, Kester et al. sc-RNA-seq dataset .
The heatmap shows the expression pattern of genes selected from Fig. 3 and Fig. 5 in the single cells studied by Baron, Kester et al. [25] using sc-RNA-seq. These cells were isolated from E11 mouse AGM and included endothelial cells (VE-Cad$^+$ Gfi1$^-$ Kit$^-$), haemogenic endothelial cells (VE-Cad$^+$ Gfi1$^+$ Kit$^-$), EHT cells (VE-Cad$^+$ Gfi1$^+$ Kit$^+$), Pre-HSC (VE-Cad$^+$ CD45$^{-/+}$). Based on hierarchical clustering (Euclidian distance), most of endothelial and haemogenic endothelial cells clustered together and correspond to CD44^{Low}Kit^{Neg} cells, some endothelial cells formed a cluster of CD44^{Neg} cells while EHT cells and Pre-HSC formed a cluster of CD44^{Low}Kit^{Pos}. The genes were grouped in the 4 indicated categories. See also Supplementary Data 7.

Supplementary Figure 10

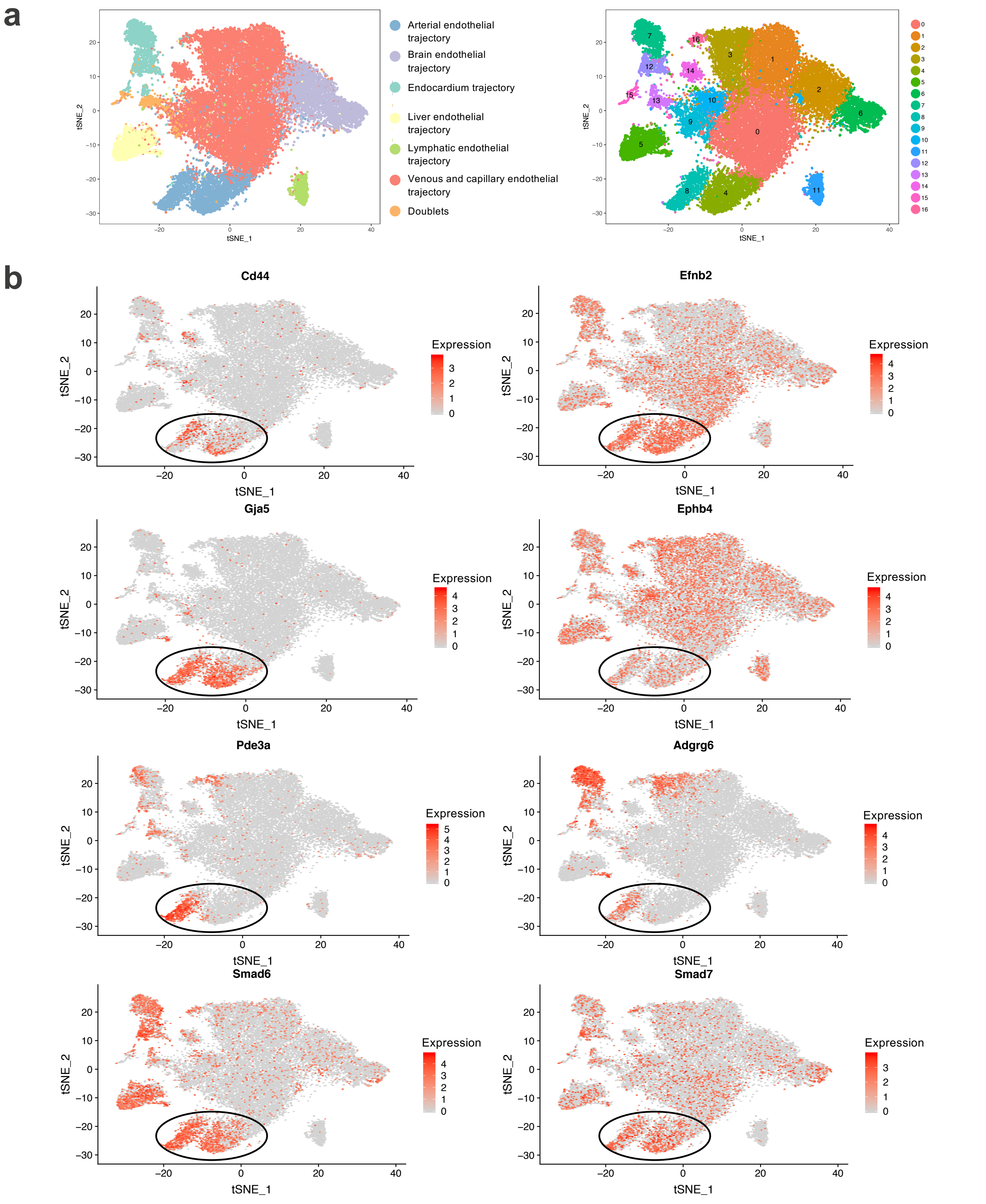

Supplementary Figure 10: Expression of *Cd44* is restricted to arterial endothelial cells between E9.5 and E13.5. (**a**) The t-SNE plots show the 26,107 endothelial cells from the Cao et al. mouse organogenesis atlas according to specific trajectories (left panel) and based on their belonging to distinct clusters (right panel). (**b**) The t-SNE plots show the expression of the indicated genes. The black ellipse highlights the cells with arterial features (co-expression of *Efnb2* and *Gja5*). The scale of gene expression is indicated next to each plot.

Supplementary Figure 11

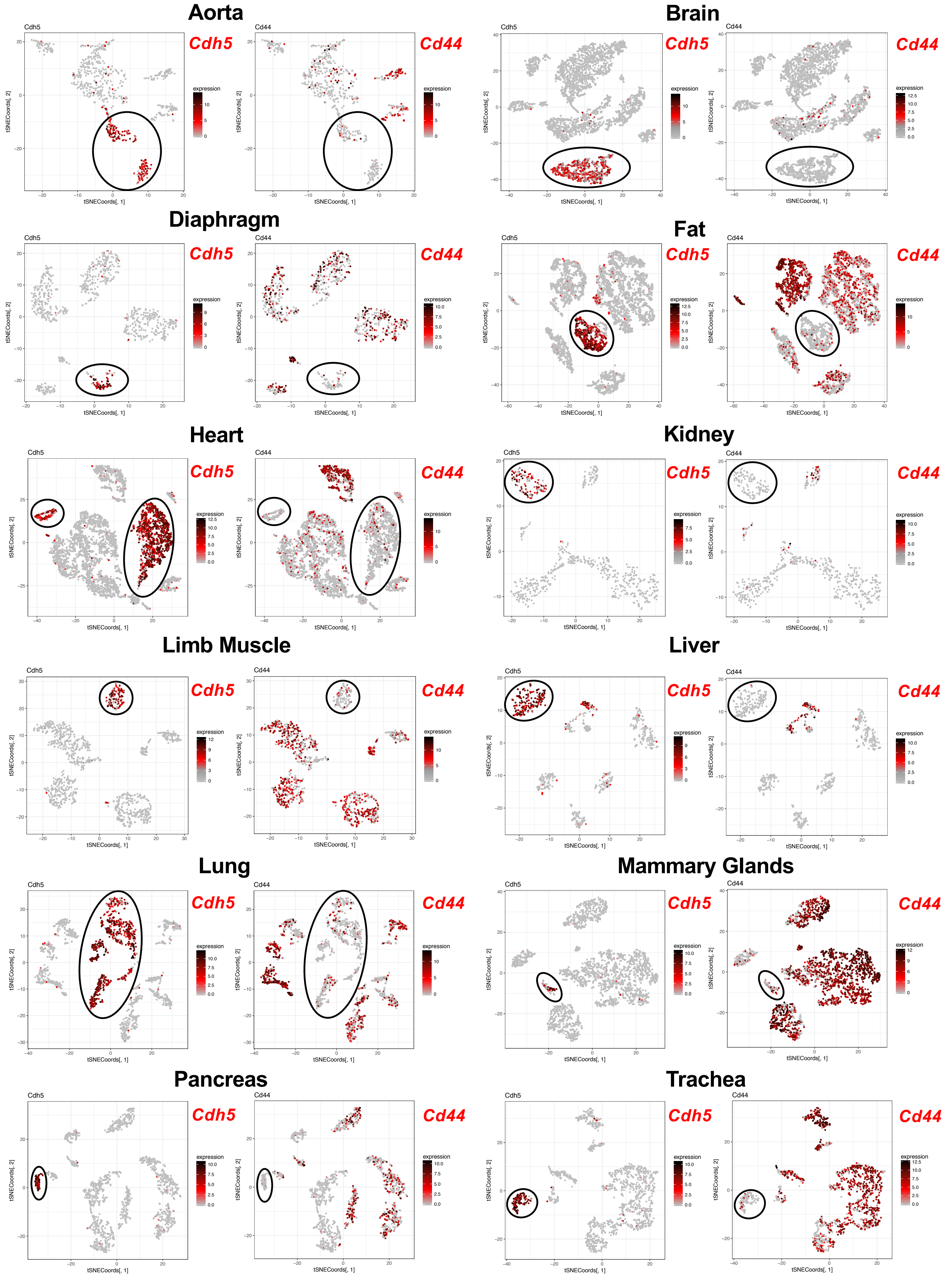

Supplementary Figure 11: Expression of *Cd44* is absent from most of adult mouse endothelial cell populations. The t-SNE plots show the expression pattern of the *Cdh5* and *Cd44* genes in endothelial cells of twelve different mouse adult organs from the Tabula Muris dataset (Single FACS-sorted cells were analysed with the Smart-Seq2 full-length transcriptome method). The scale of gene expression is indicated next to each plot.

Supplementary Figure 12

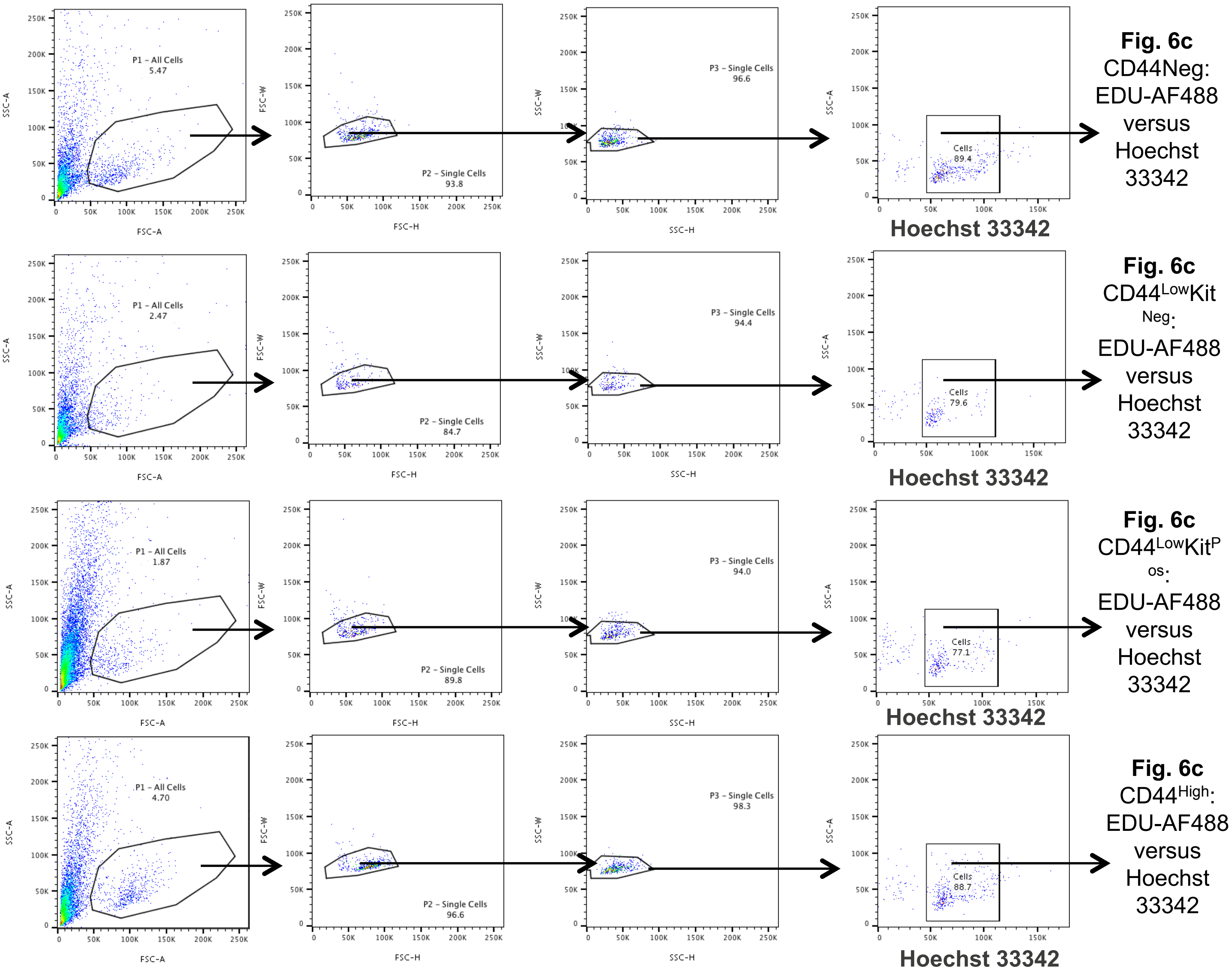

Supplementary Figure 12: Gating strategies using for FACS analysis shown in Figure 6c. From left to right: FSC-A versus SSC-A, FSC-H versus FSC-W, SSC-H versus SSC-W and SSC-A versus Hoechst 33342. The arrows indicate the cells selected from one plot to the next.

Supplementary Figure 13

a

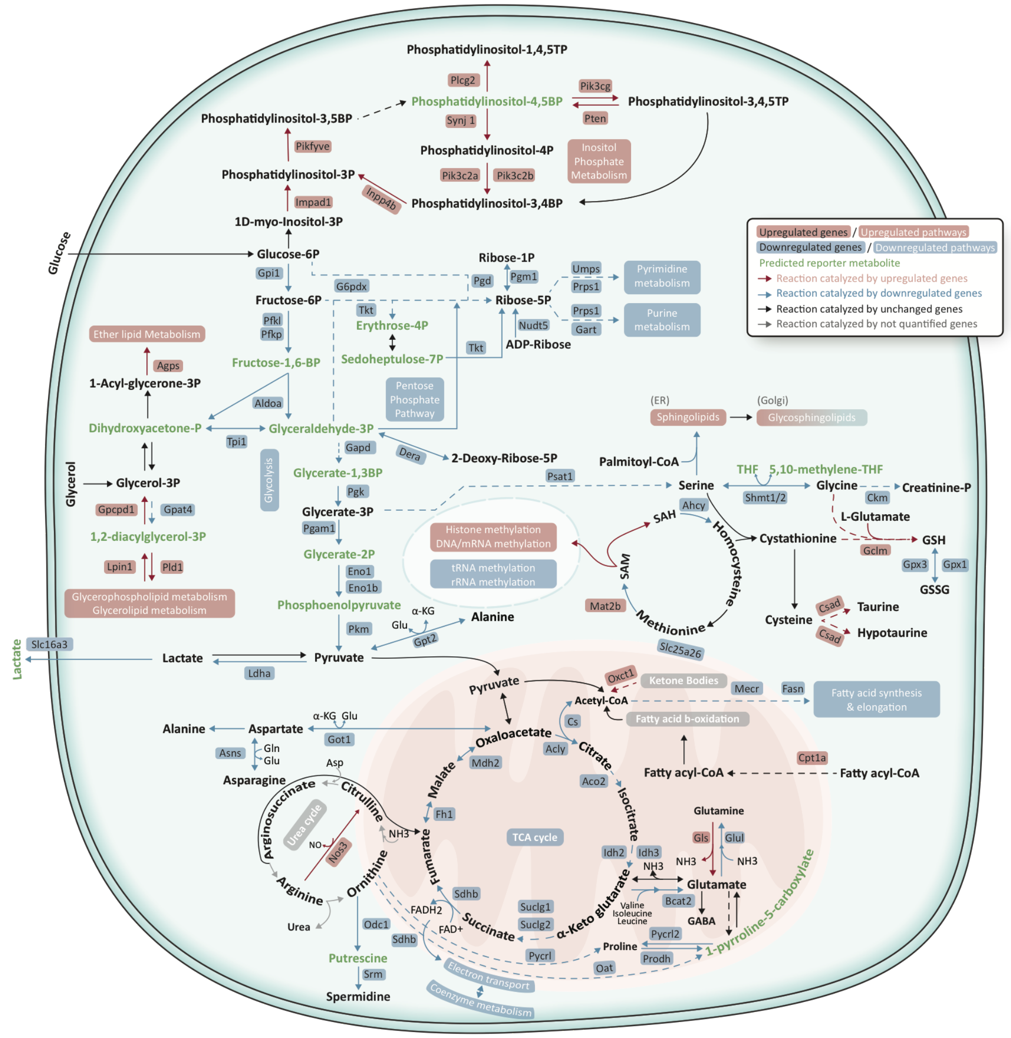

b

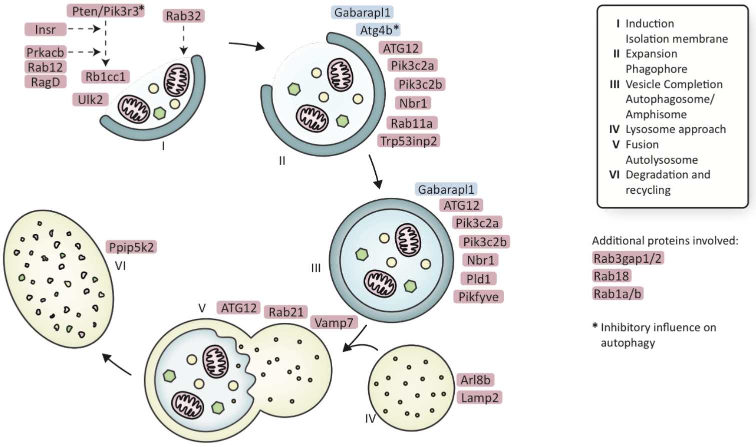

Supplementary Figure 13: CD44^Low^Kit^Neg^ endothelial cells feature altered expression of genes involved in metabolism and autophagy. **(a)** Overview of key metabolic nodes and pathways enriched in differentially expressed genes when comparing the $CD44^{Low}Kit^{Neg}$ and $CD44^{Neg}$ endothelial populations. These were selected based on reporter metabolite analysis. Pathway boxes summarize multiple genes / reporter metabolites (Supplementary Table 3). The mentioned upregulated and downregulated genes refer to the expression in the $CD44^{Low}Kit^{Neg}$ population compared to $CD44^{Neg}$. **(b)** Schematic representation of the autophagy process marking differentially expressed genes. Included are genes coding for structural as well as regulatory aspects of autophagy. The colour code is the same as in (a). See also Figure 6.

Supplementary Figure 14

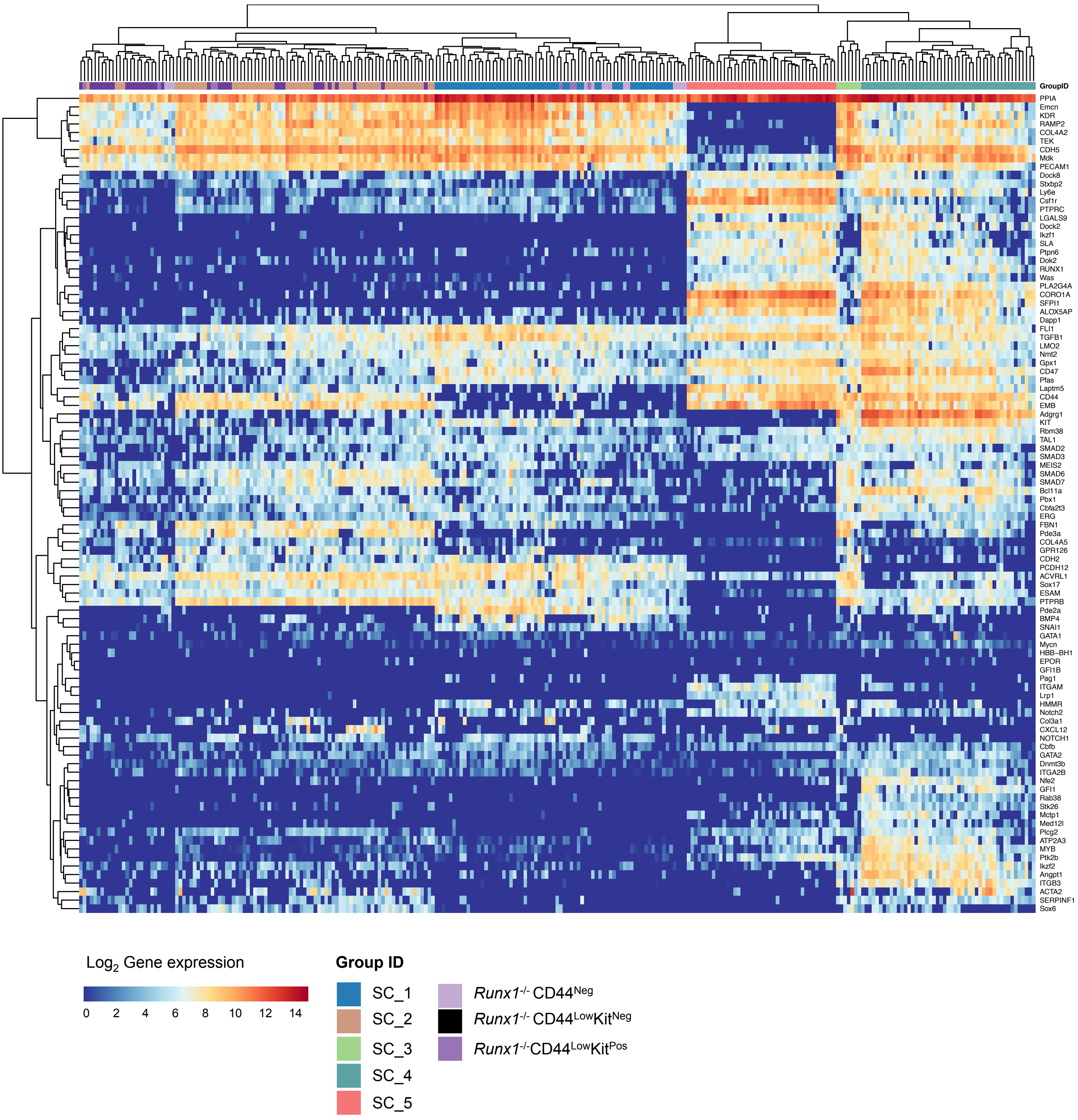

Supplementary Figure 14: Results of single cell q-RT-PCR analysis in the wild type and *Runx1*$^{-/-}$ AGM. Single cells from *Runx1*$^{-/-}$ CD44^{Neg}, *Runx1*$^{-/-}$ CD44^{Low}Kit^{Neg} and *Runx1*$^{-/-}$ CD44^{Low}Kit^{Pos} populations were isolated and tested by single-cell q-RT-PCR. The heatmap shows the result of the hierarchical clustering analysis in combination with the wild type single-cells from Figure 3a (cells were clustered by Euclidian distance and the genes by Pearson correlation). See also Figure 7 and Supplementary Data 9.

Supplementary Figure 15

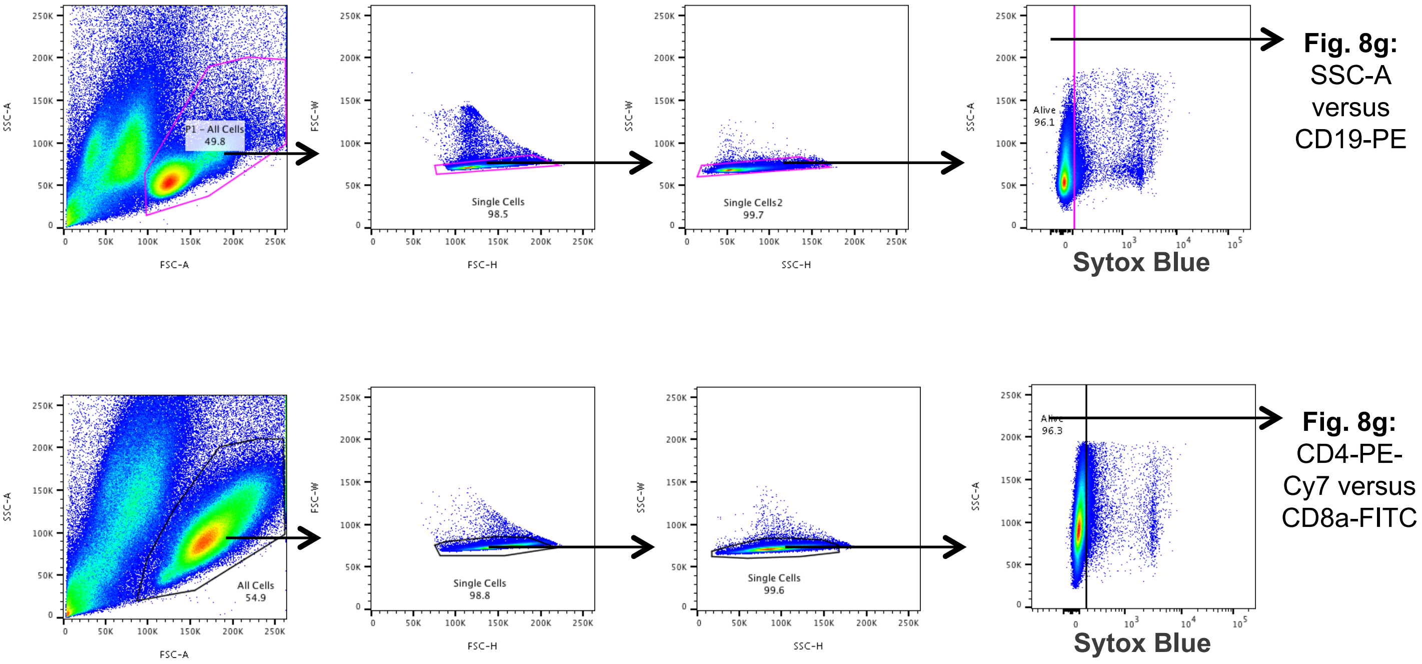

Supplementary Figure 15: Gating strategies using for FACS analysis shown in Figure 8g.
From left to right: FSC-A versus SSC-A, FSC-H versus FSC-W, SSC-H versus SSC-W and SSC-A versus dead cell dye Sytox Blue. The arrows indicate the cells selected from one plot to the next.

Supplementary Figure 16

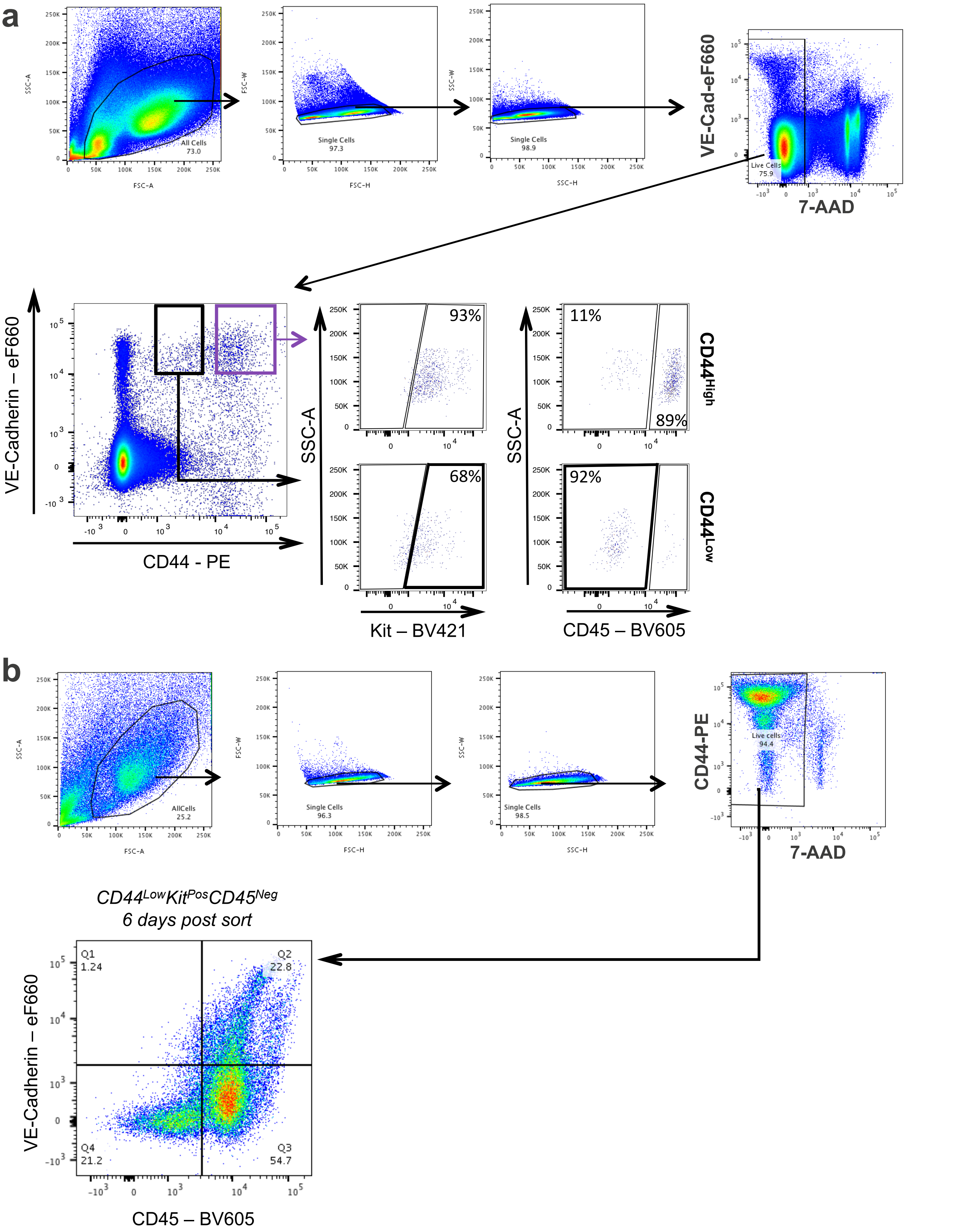

Supplementary Figure 16: CD44^{Low}Kit^{Pos} cells give rise to VE-Cad^{Pos} CD45^{Pos} cells in OP9 co-culture.
(**a**) FACS plots indicating the gating strategy to isolate VE-Cad^{Pos}CD44^{Low} Kit^{Pos}CD45^{Neg} from E10.5 AGM for OP9 co-culture. (b) FACS plots indicating the gating strategy to analyse the expression of CD45 and VE-Cad following six days of OP9 co-culture.

Supplementary Figure 17

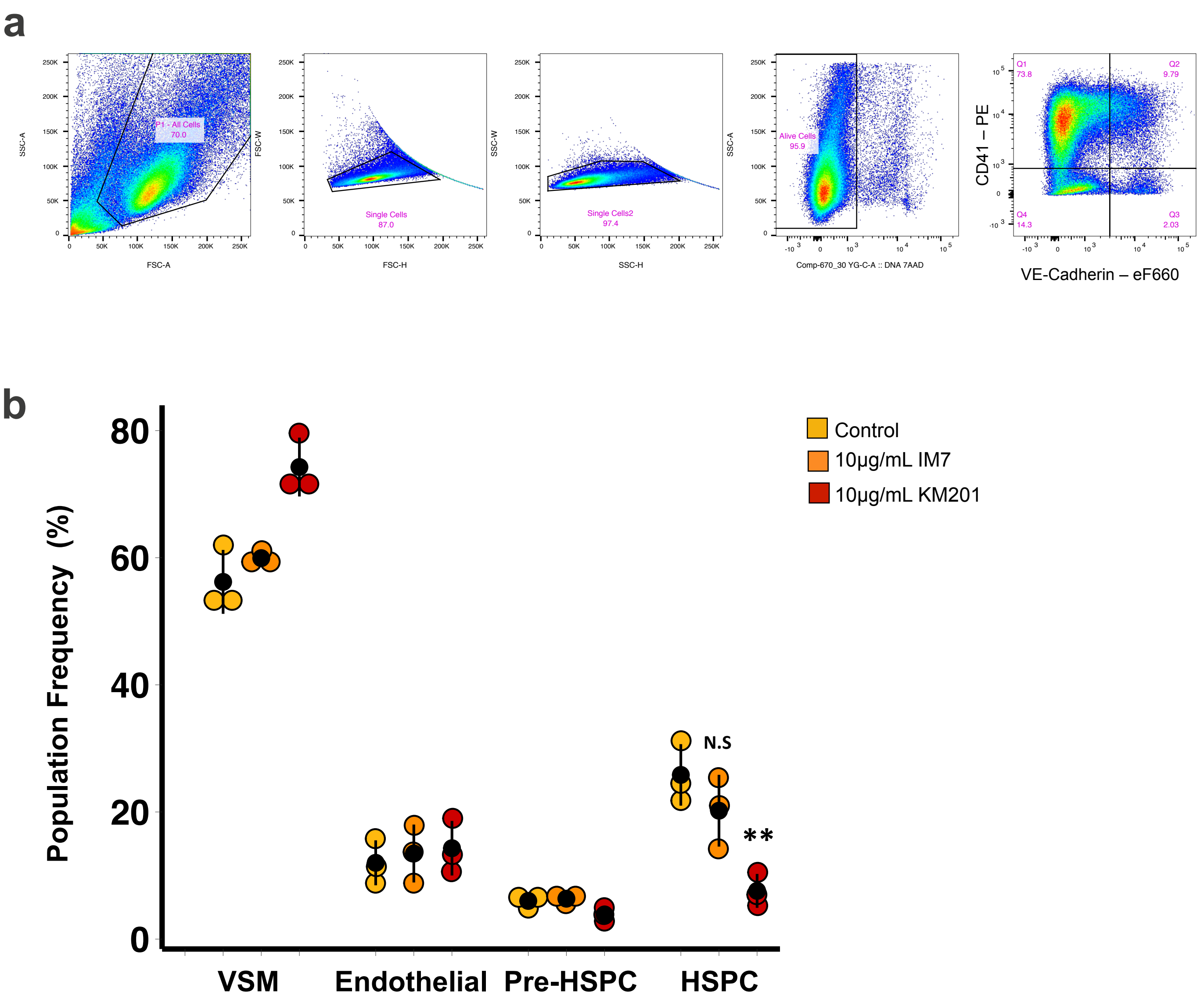

Supplementary Figure 17: Only an antibody binding close to the hyaluronan binding site on the extracellular domain of CD44 disrupts EHT in vitro. (**a**) FACS gating strategy to analyse the expression of CD41 and VE-Cad in haemangioblast culture. (**b**) Dot plot showing the population percentage for vascular smooth muscle (VSM)(VE-Cad-CD41-), endothelial cells (VE-Cad+CD41-), Pre-HSPCs (VE-Cad+CD41+) and HSPCs (VE-Cad-CD41+) after two days of haemangioblast differentiation. Treatment with KM201 disrupted EHT while IM7, another CD44 antibody, did not. N=3. Significance was determined for HSPC population using a one-way ANOVA followed by Dunnett's post hoc tests (see supplementary material). Source data are provided as a Source Data file. See also Figure 9.

Supplementary Table 1

Target protein	Gene symbol	Gene name	Bimodal expression
CD9	*Cd9*	CD9 antigen	No
CD13	*Anpep*	alanyl (membrane) aminopeptidase	No
CD19	*Cd19*	CD19 antigen	No
**CD23**	***Fcer2a***	**Fc receptor, IgE, low affinity II, alpha polypeptide**	**Yes**
CD24	*Cd24a*	CD24a antigen	No
CD29	*Itgb1*	integrin beta 1 (fibronectin receptor beta)	No
CD31	*Pecam1*	platelet/endothelial cell adhesion molecule 1	No
**CD34**	***Cd34***	**CD34 antigen**	**Yes**
CD35	*Cr2*	complement receptor 2	No
CD38	*Cd38*	CD38 antigen	No
**CD41**	***Itga2b***	**integrin alpha 2b**	**Yes**
**CD44**	***Cd44***	**CD44 antigen**	**Yes**
CD47	*Cd47*	CD47 antigen	No
**CD49d**	***Itga4***	**integrin alpha 4**	**Yes**
CD49e	*Itga5*	integrin alpha 5 (fibronectin receptor alpha)	No
**CD51**	***Itgav***	**integrin alpha V**	**Yes**
**CD54**	***Icam1***	**intercellular adhesion molecule 1**	**Yes**
**CD55**	***Cd55***	**CD55 molecule, decay accelerating factor for complement**	**Yes**
**CD61**	***Itgb3***	**integrin beta 3**	**Yes**
CD62e	*Sele*	selectin, endothelial cell	No
**CD71**	***Tfrc***	**transferrin receptor**	**Yes**
CD81	*Cd81*	CD81 antigen	No
CD93	*Cd93*	CD93 antigen	No
CD94	*Klrd1*	killer cell lectin-like receptor, subfamily D, member 1	No
CD98	*Slc3a2*	solute carrier family, member 2	No
CD102	*Icam2*	intercellular adhesion molecule 2	No
CD104	*Itgb4*	integrin beta 4	No
**CD106**	***Vcam1***	**vascular cell adhesion molecule 1**	**Yes**
**CD117**	***Kit***	**KIT proto-oncogene receptor tyrosine kinase**	**Yes**
**CD119**	***Ifngr1***	**interferon gamma receptor 1**	**Yes**
CD137	*Tnfrsf9*	tumor necrosis factor receptor superfamily, member 9	No
CD138	*Sdc1*	syndecan 1	No
CD144	*Cdh5*	cadherin 5	No
CD147	*Bsg*	basigin	No
CD200	*Cd200*	CD200 antigen	No
CD284	*Tlr4*	toll-like receptor 4	No
CD309	*Kdr*	kinase insert domain protein receptor	No
Crry/p65	*Cr1l*	complement component (3b/4b) receptor 1-like	No
**MadCam1**	***MadCam1***	**mucosal vascular addressin cell adhesion molecule 1**	**Yes**
Meca32	*Plvap*	plasmalemma vesicle associated protein	No
**PIR-A/B**	**NA**	**NA**	**Yes**
**Sca1**	***Ly6a/e***	**lymphocyte antigen 6 complex, locus A/E**	**Yes**

Supplementary Table 1: Results of the antibody screen. List of the forty-two antigens (out of 176) expressed by VE-Cad^{+} cells from day 1.5 haemangioblast culture following the antibody screen. Sixteen of these markers have a bimodal expression (indicated in bold). See also Supplementary Figure 1.

Supplementary Table 2

Gene Name	Outer forward primer	Outer reverse primer	Inner forward primer	Inner reverse primer
Acta2	aggcaccactgaaccctaag	cacagcctgaatagccacat	ccaaccgggagaaaatgac	atggcggggacattgaag
Acvrl1	cgaattgcccatcgtgacctcaa	cgtggttgttgccgatatccaggta	cgaagtcgcaatgtgctggtcaa	cgaagtcgcaatgtgctggtcaa
Adgrg1	gtggtggaggtcttcggtac	gggccgtagttattcacatcca	ctatgtgcccggctatctg	caacgccaccagagtga
Alox5ap	tgcgtaccccactttccttgt	tctctctcccagatagccgaca	gcaggactactttgcagcca	aggtacatcagtccggcgaagg
Angpt1	cagcacgaaggatgctgataa	cccgcagtgtagaacattcc	ctgtatgtgcaaatgcgctctc	atttagattggaagggccacagg
Atp2a3	cgaccattgtggctgcagtagaa	cgtcccagaattgctgtgaggaa	cgagagggcagggccatctaca	cgagagggcagggccatctaca
Bcl11a	cccgggatgagtgcagaata	ctgtgcgtgttgcaagagaa	gccccgcagggtatttgtaaa	atgcactggtgaatggctgtt
Bmp4	cagccgagccaacactgtga	tgggatgctgctgaggttga	agtttccatcacgaagaacatctgg	gaggaaacgaaaagcagagc
Cbfa2t3	aaccagcaagaggactcca	ctcccagtctttgtgctgac	gcgagagctgctggaac	agaaggacccgcagtagc
Cbfb	cccggaccagaggagcaa	cggcaggcgttctggaa	tcgagaacgaggagttcttcag	cgtgtctggcgctcct
Cd44	cccctcctgaagaagactgtaca	gctgtagcgagtaccatcacg	gtcacagacctacccaattccttcga	gctgtagcgagtaccatcacg
Cd47	cttctggacttggcctcattgt	acgtagcccagcacttgagt	tctctacggggatattaatactacttcag	tggcaatggtgaaagaggtca
Cdh2	atcaacaatgagactggggacatca	cttccatgtctgtggcttgaa	cactgtggcagctggtctgg	attaacgtatactgttgcactttctctcg
Cdh5	cgacaccatcgccaaaagagagac	cgtcttagcattctggcggttcac	cgaggatttggaatcaaatgcacatcg	cgaggatttggaatcaaatgcacatcg
Col3a1	tcttctcacccttcttcatccc	tgacatggttctggcttcca	actcttattttggcacagcagtc	tctctagactcataggactgaccaa
Col4a2	cgaccctggaagccctggattta	cgttgtcttcccttaagtcccaaca	cgatggcagggatgcctggt	cgatggcagggatgcctggt
Col4a5	cgaggactctctgtggattggcta	cgtatgaagggagcggaacgaa	cgattcatgatgcatacaagtgcagga	cgattcatgatgcatacaagtgcagga
Coro1a	tctgcgctgtcaaccccaagt	accaggggcacgttcttgtct	gctctgatctgtgaggccag	actcgtccagtcttgcctag
Csf1r	accctactcagttgccctaca	gcccagaccaaaggctgta	gtgggagttccctcggaacaa	gcctccaccaccttcccaaa
Cxcl12	tcttcgagagccacatcgcc	tgtctgttgttgttcttcagccgt	agagccaacgtcaagcatctga	ctgaagggcacagtttggagt
Dapp1	acagagtgctcagctgttcaa	ccattcatcggcttcaactcc	tttgattactcacaggaacgagtaa	cgtctttgcacagagataaaatgt
Dnmt3b	ttggtggaagcccatgca	ccttggggcgggtataattca	atgatctctctaacgtcaatcctg	gcaagtggtaaaactcgaagaag
Dock2	gtaccactcagtcgtctactacc	gagcggtgtcgaaacatgaa	aagtcaaacagccccgatg	cctcaaatggatcctctgcat
Dock8	ggattctggacctgctgttca	tcttccagcttggccttgac	tctgtgtctcctgctttgaata	tctcttgacttctgcaggac
Dok2	agccaggcacacagctata	actcaaagttgccctctcca	tgactggccctacaggtttct	gtctgccagcctcaaaggaaa
Emb	cctggttacccgctcctccttct	cgccatcatttcttctcgaacaggt	atgtcttctggcggcgac	acttgtaaaagttggatctgtggga
Emcn	taggaagattgcaaccactcca	attcgatacaaacccaccagagta	caaccaccccctcctattcca	gttataacaaccagcgcgataacca
Epor	tgaagtggacgtgtcggcag	acagcgaaggtgtagcgcgt	caaccgggcaggagggaca	ccgccccgcaggttgctcagaa
Erg	tcccgaagctacgcaaagaa	tttggactgaggggtgaggtg	tacaactaggccagatttaccttatga	tgtggccggtccaggctgat
Esam	cgaagtctatgtctgcaaggctcaa	cgtcccaacaaaagtgcccacaa	cgacagagtgggctttgccaagt	cgacagagtgggctttgccaagt
Fbn1	acgtggcggggaatgtacaaaca	cgtcagagctgtgtagcagtaacca	cgactgtcagcagctacttctgcaaat	cgactgtcagcagctacttctgcaaat
Fli1	tgctgttgtcgcacctcagtt	ttccttgacattcagtcgtgagga	ctcagggaaagttcactgctggccta	tggtctgtatgggaggttgtg
Gata1	cctgtgcaatgcctgtggct	tgcctgcccgtttgctgacaa	gtatcacaagatgaatggtcagaacc	cattcgcttcttgggccggatg
Gata2	aagcaaggctcgctcctg	cacaggcattgcacaggtagt	cagaaggccgggagtgtgtc	gcccgtgccatctcgt
Gfi1	cgagagatgtgcggcaagacc	cgtagcgtggatgacctcttgaa	cgagtgagcctggagcaacacaa	cgagtgagcctggagcaacacaa
Gfi1b	cgatggacacttaccactgtgtca	cgtaggttttgccacagacatcac	cgaagtgcaacaaggtgttctcc	cgaagtgcaacaaggtgttctcc
Gpr126	cgactgtgcagccacttcactca	cgtggcagatattccgcacccaata	cgatggagttctgatggatcttcc	cgatggagttctgatggatcttcc
Gpx1	cgggactacaccgagatgaa	cggacgtacttgagggaattca	cagaagcgtctgggacct	tcttcattcttgccattctcctg
Hbb-Bh1	gagctgcactgtgacaagcttca	ggggtgaattccttggcaaaa	tggatcctgagaacttcaagc	gagtagaaaggacaatcaccaaca
Hmmr	tgcacagctacttggtcacca	agctgagatcggagttttgacacct	ggtcaccagaacctaaagcaaa	cacctccgatttgagttggct
Ikzf1	gcaagcaatgtcgccaaac	tccatcacgtgggatgtca	gagacaagtgcctgtcaga	tcatatcctccttctcatagttgg
Ikzf2	aagaagggacgctctcaca	acagcgttccttgtgttcc	gacacctcaggacccattctg	tgagctgcgctgcttgta
Itga2b	ttccaaccagcgcttcacct	tgctcggatccccatcaaac	cgacaacagcaacccagtgttt	gcccacggctaccgaatatc
Itgam	agcaggggtcattcgctacg	cagctggcttagatgcgatgg	attggggtgggaaatgccttc	gtcgagctctctgcgggact
Itgb3	tcctccagctcattgttgatgc	aggcaggtggcattgaagga	acgggaaaatccgctctaaa	agtgacagttcttccggcaggt
Kdr	tgtggggcttgatttcacctg	tcgccacagtcccaggaaag	cactctccaccttcaaagtctcatca	tttcacatcccggtttacaatcttc
Kit	ctggctctggacctggatga	cctggctgccaaatctctgtg	tgctgagcttctcctaccaggtg	atacaattcttggaggcgaggaa
Laptm5	atgacactgcagctgctagac	cctcctggcttgggaggtaa	ctgtttgagtatcctgaccctgtg	catgtggttcatggacttgaagttg
Lgals9	tggtgcagaggtcagagttca	aagcagccggagacagcgat	aggtgatggtgaacaagaaattct	ggtagggtacgcggtgttgg
Lmo2	tcggccatcgaaaggaagag	gcggtcccctatgttctgct	ctggacccgtctgaggaacc	gcagccaccacatgtcagca
Lrp1	tgggtggatgccttctatgac	aggtagttgccatggtgaca	aattgagaccatactgctcaatgg	caggccgaaggcatgattc
Ly6e	ctttggcttgcgaaccttca	acatgagaagcacatcagggaa	gcagatgtctgccacttccaa	gaacttgctccatgcccagaa
Mctp1	cctaaatcctgtgtgggaggaa	aaggctgagcccataaagtca	aaggcttgcgtgctcatt	tcttgcagtccaaaatcatagtca
Mdk	cctgtcagctctgtcaatcac	tcagggtggggagaacaaaa	tgtcctctcacgcccacac	agggtatggggaggctcact
Med12l	ctttgtgtgcaacaccctca	ccactccgaactcagaactgta	aatgtgtgcatgggacaccag	caggcagttagctcagaggaga
Meis2	cgacccgtacccttcagaagaacagaa	cgttggtcaatcatgggctgcacta	cgagaaacagttagcgcaagacac	cgagaaacagttagcgcaagacac
Myb	cgagtggcagaaagtgctgaacc	cgttgcttggcaataacagaccaac	cgacatcaaaggtccctggaccaaa	cgacatcaaaggtccctggaccaaa
Mycn	gagccttcgaattgggctac	catgcagtcctgaaggatgac	agatgctgctgccggaggccgac	agtgaggccacccaggccccc
Nfe2	tcctcagcagaacaggaaca	tgaggctcaaaagatgtctcac	ggttatcacagctgcctgtt	tttagaccctgcagctcagta
Nmt2	gccacaaaacgcagatacca	aagaatatggttcctggcggata	tgggacacacagccagt	ttgtctttgtctggttcaattgc
Notch1	cgaccaaccctgtcaacggcaaa	cgtatttgcctgcgtgctcacaa	cgatgcccctcggggtaca	cgatgcccctcggggtaca
Notch2	agctgctactcacaggtgaa	cctcacagttgacaccaacc	cgagtgcctgagcaatcc	catcgcagaggcacttatagc
Pag1	acaagtctcgggaagaagacc	gcagattctggccctttcatac	cgactcttacagaagaggagatctcag	ccaggtttgtgtgccgactg
Pbx1	caggacatcggggacattttaca	cacattaaacaaggcaggcttca	aattatgaccatcacagaccagagtt	ttctgtggcagtttaaagcatgtt
Pcdh12	cgatggctgcttttgcggaac	tcgtggtttggtttgggctggaa	cgaggaacccggtggagga	cgaggaacccggtggagga
Pde2a	ctgtcctgctacaggagatcatca	ccacaacgccaccatcgaa	cggaagccagaaacctca	ccaccagctcgttctgat
Pde3a	tacacgctgtgtggtatctca	atcccatgtgtccgtgtgta	attcctggcctcccaagt	tccactgtcagaatcggagt
Pecam1	tgcggtggttgtcattggag	ctggacatctccacgggttt	gtcatcgccaccttaatagttgcag	tgtttggccttggctttcctc
Pfas	caagagctgcagcggaac	cgctagcttcttcccatcca	ccagtacagtggaggtctttga	catggagctggcccttga
Pla2g4a	cgtgtggaatgagacctttgagt	agggaatgtagctgtgcctagggt	acctttgagttcattttggatcct	agtgtgatctccaaaacattttcctga
Plcg2	agcatgcgctctgagaagta	ggcaatactccgccctagttta	tgatccgatgcccctggag	ggtggcgtgcaccaagaa
Ppia	cgaccgactgtggacagctctaa	cgtagtgagagcagagattacaggac	cgatttcttttgacttgcgggcatt	cgatttcttttgacttgcgggcatt
Ptk2b	agaaagactgtacccaggacaac	tccaggtgggttcctcttca	aaggagaagttcatgagtgagg	tgccaatcagcttcacgat
Ptpn6	tgactaccagagaggtggagaa	ctgctgtgtcatgctcccta	aggccggaacaaatgtgtc	tgttggtcacagagtagagacc
Ptprb	acgccaagagcggcaattatgca	cgttgcacccaggacacctttaa	cgaccactccttcaccgaggaa	cgaccactccttcaccgaggaa
Ptprc	ggcttcaaggaacccaggaaata	tgacaataactgtggccttttgctc	attgctgcacaagggccccgggatg	cagatcatcctccagaagtcatcaa
Rab38	gcgctgaaggtgctcca	ccccatagcttcccggtaataa	gtggtgcgcttgcagctctg	actcttgtcatgtttccaaatc
Ramp2	tcccactgaggacagccttg	tccttgacagagtccatgcaa	tcaaaagggaagatggaagactacga	tcttgtactcataccagcaaggtaggaca
Rbm38	gctgatagggcttgcaaagac	gctgcacaccaacagcaa	cctatcatcgatggtcgcaagg	agcccgtctgtaagctcctag
Runx1	cgaactactcggcagaactgagaa	cgtacggtgatggtcagagtgaa	cgaatgctaccgcggccatg	cgaatgctaccgcggccatg
Serpinf1	agaacgtccccagcagctct	tgccagcttgttcacagggacc	agcagctctgagggctccc	tcctcctcctccacgggct
Sla	cgacgaatcttccgtcttcccaac	tcgggtgagcacacagcatagac	cgaactggtactacatctcaccaagg	cgaactggtactacatctcaccaagg
Smad2	tgctctccaacgttaaccgaaa	tcagcaaacacttccccacct	gccactgtagaaatgacaagaagaca	tgtaatacaagcgcactccccttc
Smad3	ccaatgtcaaccggaatgcag	tgaggcactccgcaaagacc	cgtggaacttacaaggcgaca	cccctccgatgtagtagagc
Smad6	ttctcggctgtctcctcctgac	ttcacccggagcagtgatga	gtacaagccactggatctgtccgatt	ggagttggtggcctcggttt
Smad7	ggaagatcaaccccgagctg	tgagaaaatccattgggtatctgga	tgtgctgcaacccccatcac	aaggaggagggggagactcta
Snai1	cgatctgcacgacctgtggaaa	cgtgagcggtcagcaaaagca	cgactctaggccctggctgctt	cgactctaggccctggctgctt
Sox17	cgagatgggtcttccctacc	tcggggtagttgcaatagtagac	ggacacgactgcggagtgaa	gtcggacaccacggaggaaat
Sox6	tcaacctgccaaacaaaagca	ctgtgtcctggtccccaaa	gcatccccagccccattg	cagggcaggagagttgagact
Spi1	gtgggcagcgatggagaaag	tgcagctctgtgaagtggttctc	atagcgatcactactgggatttctcc	gggaagttctcaaactcgttgttg
Stk26	agcagcgcgaagtagca	gccttttccaatgcgctctaa	ccaccatggcccactca	tttgtgaacagttcttctggatctg
Stxbp2	aaacggagagaacccattcc	gggctgctttgtaggtgaa	gcttggaggcaatttatttgctgag	ggttggtgttccctggaagtc
Tal1	accggatgccttccccatgtt	gcgccgcactactttggtgt	ccaacaacaaccgggtgaaga	aggaccatcagaaatctccatctca
Tek	tccaaaggagaatggctcagg	tccggattgtttttggccttc	ttccagaacgtgagagaagaacca	tgttaagggccagagttcctga
Tgfb1	acccccactgatacgcctga	gcagtgagcgctgaatcgaa	tggctgtcttttgacgtcactg	gccctgtattccgtctccttgg
Was	cgttcagcagaacattccttcc	agtgccaggtagagctgaac	aacctcctccaggaccatgaa	ctgtggtagccagtgtccag

Supplementary Table 2: List of primers for single-cell q-RT-PCR. These primers were used to detect the genes shown in Fig. 3, Fig. 4, Supplementary Fig. 5, Supplementary Fig. 7 and Supplementary Fig. 14.

Supplementary Table 3

Antibody	Reference	Final concentration
Anti-*Mouse* CD19 PE	eBiosciences 12-0191-81	1 μg/mL
Anti-*Mouse* CD4 PE-Cy7	BD Biosciences 561099	0.25 μg/mL
anti-*Mouse* CD41-PE	eBiosciences 12-0411-82	0.5 μg/mL
Anti-*Mouse* CD43 PerCP-Cy5-5	BD Biosciences 562865	4 μg/mL
Anti-*Mouse* CD44	Abcam ab157107	2 μg/mL
Anti-*Mouse* CD44	Abcam ab25340 (KM207)	5 μg/mL and 10 μg/mL
Anti-*Mouse* CD44	BD Biosciences 550538 (IM7)	10 μg/mL
anti-*Mouse* CD44-PE	BD Biosciences 553134	0.16 μg/mL
Anti-*Mouse* CD45 BV605	BD Biosciences 563053	2 μg/mL
Anti-*Mouse* CD45-FITC	BD Biosciences 553079	5 μg/mL
Anti-*Mouse* CD51-Biotin	BD Biosciences 551380	2.5 μg/mL
Anti-*Mouse* CD61-PE	BD Biosciences 553347	1 μg/mL
Anti-*Mouse* CD8a-Alexa Fluor 488	BD Biosciences 557668	5 μg/mL
Anti-*Mouse* CD93-PE	BD Biosciences 558039	1 μg/mL
Anti-*Mouse* Flk1-APC	eBiosciences 17-5821-81	6 μg/mL
Anti-*Mouse* Kit-BV421	BD Biosciences 562609	1 μg/mL
Anti-*Mouse* Ly-6A/E (Sca1)-PE	BD Biosciences 561076	1 μg/mL
Anti-*Mouse* MAdCAM-1-Biotin	BD Biosciences 553808	2.5 μg/mL
Anti-*Mouse* VE-Cadherin	eBiosciences 14-1441-81	2.5 μg/mL
anti-*Mouse* VE-Cadherin-eFluor660	eBiosciences 50-1441-82	1 μg/mL
Anti-*Rabbit*-Alexa Fluor 488	ThermoFisher Scientific A-21206	4 μg/mL
Anti-*Rat*-Alexa Fluor 568	ThermoFisher Scientific A-11077	4 μg/mL
Mouse Cell Surface Marker Screening Panel (176 antibodies)	BD Biosciences 562208	Concentrations of antibodies are specified in the product datasheet.
Streptavidin PE-Cy7	eBioscience 25-4317	0.66 μg/mL

Supplementary Table 3: List of antibodies used in this study. Name, product reference and final concentration of antibodies are indicated.

Supplementary Table 4

Populations	E9.5	E10	E11
VE-Cad^{Pos} CD44^{Neg} (CD44^{Neg})	4 x 25-cells	3 x 25-cells	3 x 25-cells
VE-Cad^{Pos} CD44^{Low} Kit^{Neg} (CD44^{Low} Kit^{Neg})	1 x 25-cells	2 x 25-cells	3 x 25-cells
VE-Cad^{Po}s CD44^{Low} Kit^{Pos} (CD44^{Low} Kit^{Pos})	1 x 25-cells	2 x 25-cells	Not done
VE-Cad^{Neg} CD44^{High} (CD44^{High})	Not done	Not done	3 x 25-cells

Supplementary Table 4: List of samples used for bulk RNA sequencing. The phenotype of populations, time points, and number of samples are indicated.
