# Supplementary material for: Single-cell transcriptomics identifies CD44 as a marker and regulator of endothelial to haematopoietic transition
Source: Nat Commun. 2020 Jan 29;11:586. doi: 10.1038/s41467-019-14171-5 (PMC6989687; doi:10.1038/s41467-019-14171-5)
Supplement: Supplementary file 3 — Reporting Summary [file 41467_2019_14171_MOESM3_ESM.pdf]

nature research

Corresponding author(s): Christophe Lancrin

Last updated by author(s): Dec 2, 2019

Reporting Summary

Nature Research wishes to improve the reproducibility of the work that we publish. This form provides structure for consistency and transparency in reporting. For further information on Nature Research policies, seeAuthors & Referees and theEditorial Policy Checklist .

Statistics

For all statistical analyses, confirm that the following items are present in the figure legend, table legend, main text, or Methods section.

n/a	Confirmed	
☐	☒	The exact sample size (*n*) for each experimental group/condition, given as a discrete number and unit of measurement
☐	☒	A statement on whether measurements were taken from distinct samples or whether the same sample was measured repeatedly
☐	☒	The statistical test(s) used AND whether they are one- or two-sided<br>*Only common tests should be described solely by name; describe more complex techniques in the Methods section.*
☒	☐	A description of all covariates tested
☐	☒	A description of any assumptions or corrections, such as tests of normality and adjustment for multiple comparisons
☐	☒	A full description of the statistical parameters including central tendency (e.g. means) or other basic estimates (e.g. regression coefficient) AND variation (e.g. standard deviation) or associated estimates of uncertainty (e.g. confidence intervals)
☐	☒	For null hypothesis testing, the test statistic (e.g. *F*, *t*, *r*) with confidence intervals, effect sizes, degrees of freedom and *P* value noted<br>*Give P values as exact values whenever suitable.*
☒	☐	For Bayesian analysis, information on the choice of priors and Markov chain Monte Carlo settings
☒	☐	For hierarchical and complex designs, identification of the appropriate level for tests and full reporting of outcomes
☒	☐	Estimates of effect sizes (e.g. Cohen's *d*, Pearson's *r*), indicating how they were calculated

Our web collection on statistics for biologists contains articles on many of the points above.

Software and code

Policy information about availability of computer code

Data collection	FACS data was collected using FACS Diva software (v 8.0.2). Bright field microscopy images were captured using Leica Application Systems version 3.80. Fluorescence microscopy images were captured with Leica Application Suite Advanced Fluorescence software (2.7.9723.3). Single-cell qPCR data was acquired using the Fluidigm Real-Time PCR Analysis software version 4.1.2. The single-cell RNA sequencing experiment was performed using a Fluidigm C1 instrument and sequenced on an Illumina Hiseq2000 with corresponding Illumina software. The bulk RNA sequencing experiment was sequenced using an Illuminer Next Seq sequencer with corresponding Illumina software.
Data analysis	FACS data was analysed using FlowJo version 10.4.2. Single-cell qPCR data was analysed using R version 3.3.1 and Rstudio version 0.99.903. Initial analysis of qPCR data was performed using the SINGuLAR analysis toolkit by Fluidigm (version 3.5) in R and data visualisation was performed with pheatmap and Rtsne packages. Single-cell RNA sequencing transcripts were quantified using Salmon, an open source software and further analysed with python using the scikit-learn package. Bulk sequencing data was analysed with the aid of the EMBL Galaxy tools (galaxy.embl.de) - specifically, FASTX for adaptor clipping, RNA STAR for mapping and htseq-count for obtaining raw gene expression counts. The R software was then used to generate heatmaps and tSNE plots using the DESeq2, Scater, Biobase, Seurat, pHeatmap packages and the CONCLUS analysis pipeline (https://github.com/lancrinlab/CONCLUS).

For manuscripts utilizing custom algorithms or software that are central to the research but not yet described in published literature, software must be made available to editors/reviewers. We strongly encourage code deposition in a community repository (e.g. GitHub). See the Nature Research guidelines for submitting code & software for further information.

Data

Policy information about availability of data

All manuscripts must include a data availability statement. This statement should provide the following information, where applicable:

- Accession codes, unique identifiers, or web links for publicly available datasets
- A list of figures that have associated raw data
- A description of any restrictions on data availability

All the expression data (sc-q-RT-PCR, sc-RNA-seq and bulk RNA-seq) supporting the results reported in the article can be found in Supplementary Files 1, 2, 3, 4, 5, 6, 7 and 8. Additionally, the sc-RNA-seq raw data is accessible from the ArrayExpress respository (E-MTAB-6987) and the bulk RNA-seq raw data was deposited at the NCBI Gene Expression Omnibus (GSE128971). All raw FACS produced in the current study is available from the corresponding author upon reasonable request. All other data generated for this study is included in this published article or its supplementary files.

Field-specific reporting

Please select the one below that is the best fit for your research. If you are not sure, read the appropriate sections before making your selection.

☐ Life sciences ☐ Behavioural & social sciences ☐ Ecological, evolutionary & environmental sciences

For a reference copy of the document with all sections, see nature.com/documents/nr-reporting-summary-flat.pdf

Life sciences study design

All studies must disclose on these points even when the disclosure is negative.

Sample size	The sample sizes for single-cell transcriptome analysis were chosen in order to maximize our ability to detect rare populations from mouse embryos. A step of enrichment using flow cytometry and specific cellular markers allowed us to isolate between 10 and 50 cells for each population that we were analysing per experiment.
Data exclusions	Eighteen single cells were excluded from our sc-RNAseq experiment which did not meet our technical requirements of having >500,000 mapped reads and <30% mitochondrial gene content.
Replication	The original single cell RNAsequencing experiment was performed only once from a pool of mouse embryos. FACS analysis, single-cell qPCR, OP9 functional studies and bulk RNA sequencing was performed on multiple biological replicates – pooled samples of mouse embryos. In vitro haemangioblast experiments were performed on multiple different batches of ES cells differentiated independently.
Randomization	N/A – No randomization was performed as this was not a case/control study
Blinding	*Describe whether the investigators were blinded to group allocation during data collection and/or analysis. If blinding was not possible, describe why OR explain why blinding was not relevant to your study.*

Behavioural & social sciences study design

All studies must disclose on these points even when the disclosure is negative.

Study description	*Briefly describe the study type including whether data are quantitative, qualitative, or mixed-methods (e.g. qualitative cross-sectional, quantitative experimental, mixed-methods case study).*
Research sample	*State the research sample (e.g. Harvard university undergraduates, villagers in rural India) and provide relevant demographic information (e.g. age, sex) and indicate whether the sample is representative. Provide a rationale for the study sample chosen. For studies involving existing datasets, please describe the dataset and source.*
Sampling strategy	*Describe the sampling procedure (e.g. random, snowball, stratified, convenience). Describe the statistical methods that were used to predetermine sample size OR if no sample-size calculation was performed, describe how sample sizes were chosen and provide a rationale for why these sample sizes are sufficient. For qualitative data, please indicate whether data saturation was considered, and what criteria were used to decide that no further sampling was needed.*
Data collection	*Provide details about the data collection procedure, including the instruments or devices used to record the data (e.g. pen and paper, computer, eye tracker, video or audio equipment) whether anyone was present besides the participant(s) and the researcher, and whether the researcher was blind to experimental condition and/or the study hypothesis during data collection.*
Timing	*Indicate the start and stop dates of data collection. If there is a gap between collection periods, state the dates for each sample cohort.*
Data exclusions	*If no data were excluded from the analyses, state so OR if data were excluded, provide the exact number of exclusions and the rationale behind them, indicating whether exclusion criteria were pre-established.*

Non-participation	*State how many participants dropped out/declined participation and the reason(s) given OR provide response rate OR state that no participants dropped out/declined participation.*
Randomization	*If participants were not allocated into experimental groups, state so OR describe how participants were allocated to groups, and if allocation was not random, describe how covariates were controlled.*

Ecological, evolutionary & environmental sciences study design

All studies must disclose on these points even when the disclosure is negative.

Study description	*Briefly describe the study. For quantitative data include treatment factors and interactions, design structure (e.g. factorial, nested, hierarchical), nature and number of experimental units and replicates.*
Research sample	*Describe the research sample (e.g. a group of tagged Passer domesticus, all Stenocereus thurberi within Organ Pipe Cactus National Monument), and provide a rationale for the sample choice. When relevant, describe the organism taxa, source, sex, age range and any manipulations. State what population the sample is meant to represent when applicable. For studies involving existing datasets, describe the data and its source.*
Sampling strategy	*Note the sampling procedure. Describe the statistical methods that were used to predetermine sample size OR if no sample-size calculation was performed, describe how sample sizes were chosen and provide a rationale for why these sample sizes are sufficient.*
Data collection	*Describe the data collection procedure, including who recorded the data and how.*
Timing and spatial scale	*Indicate the start and stop dates of data collection, noting the frequency and periodicity of sampling and providing a rationale for these choices. If there is a gap between collection periods, state the dates for each sample cohort. Specify the spatial scale from which the data are taken*
Data exclusions	*If no data were excluded from the analyses, state so OR if data were excluded, describe the exclusions and the rationale behind them, indicating whether exclusion criteria were pre-established.*
Reproducibility	*Describe the measures taken to verify the reproducibility of experimental findings. For each experiment, note whether any attempts to repeat the experiment failed OR state that all attempts to repeat the experiment were successful.*
Randomization	*Describe how samples/organisms/participants were allocated into groups. If allocation was not random, describe how covariates were controlled. If this is not relevant to your study, explain why.*
Blinding	*Describe the extent of blinding used during data acquisition and analysis. If blinding was not possible, describe why OR explain why blinding was not relevant to your study.*

Did the study involve field work? ☐ Yes ☐ No

Field work, collection and transport

Field conditions	*Describe the study conditions for field work, providing relevant parameters (e.g. temperature, rainfall).*
Location	*State the location of the sampling or experiment, providing relevant parameters (e.g. latitude and longitude, elevation, water depth).*
Access and import/export	*Describe the efforts you have made to access habitats and to collect and import/export your samples in a responsible manner and in compliance with local, national and international laws, noting any permits that were obtained (give the name of the issuing authority, the date of issue, and any identifying information).*
Disturbance	*Describe any disturbance caused by the study and how it was minimized.*

Reporting for specific materials, systems and methods

We require information from authors about some types of materials, experimental systems and methods used in many studies. Here, indicate whether each material, system or method listed is relevant to your study. If you are not sure if a list item applies to your research, read the appropriate section before selecting a response.

Materials & experimental systems

n/a	Involved in the study	
	x	Antibodies
	x	Eukaryotic cell lines
x		Palaeontology
	x	Animals and other organisms
x		Human research participants
x		Clinical data

Methods

n/a	Involved in the study	
x		ChIP-seq
	x	Flow cytometry
x		MRI-based neuroimaging

Antibodies

Antibodies used

Antibodies used in flow cytometry
Flk1-APC antibody (1:200; eBioscience cat#17-5821-81, clone Avas12a1, lot#1998335); VE-Cadherin-AF660 antibody (1:200; eBioscience cat#50-1441-82, clone BV13, lot# 4347801); CD41-PE antibody (1:400; eBiosciences cat#12-0411-82, clone MWReg30, lot#1937683); CD41-FITC antibody (1:100; BD Biosciences cat#561849, MWReg30, lot#4275502); CD117-BV421 antibody (1:200, BD Biosciences cat#562609, clone 2B8, lot#8162767); CD44-PE antibody (1:2400, BD Biosciences cat#5096562, clone IM7, lot#5096562); CD45-BV605 antibody (1:100, BD Biosciences cat#563053, clone 30-F11, lot#7250764); CD43-PerCP-Cy5.5 antibody (1:50, BD Biosciences cat#562865, clone S7, lot#5176749); CD19-PE antibody (1:200, eBioscience cat#12-0191-81, clone MB19.1, lot#4299785); CD4-PEcy7 antibody (1:800, BD Biosciences cat#561099, clone RM4-5, lot#5205548); CD8a-AF488 antibody (1:100, BD Biosciences cat#557668, clone 53.6.7, lot#7130575); CD61-PE antibody (1:200, BD Biosciences cat#553347, clone 2C9.G2); CD93-PE antibody (1:200, BD Biosciences cat#558039, clone AA4.1); Ly-6A/E(Sca1)-PE antibody (1:200, BD Biosciences cat#561076, clone E13-161.7);MAdCAM-BIO antibody (1:200, BD Biosciences cat#553808, clone MECA-89);CD51-BIO antibody (1:200, BD Biosciences cat#551380, clone RMV-7); Mouse Cell Surface Marker Screening Panel (BD Biosciences cat#562208).

Unconjugated antibodies
Polyclonal Rabbit Anti-CD44 antibody (1:500, Abcam cat#ab157107, lot#GR271761-1); Monoclonal Rat Anti-VE-Cadherin antibody (1:200, eBioscience cat#14-1441-81, clone eBioBV13, lot#E04061-1631); Monoclonal Rat Anti-CD44 antibody (10µg/mL, Abcam Cat#ab25340, clone KM201, lot#GR262641-3).

Validation

Flow cytometry antibodies were tested by both eBioscience and BD biosciences in comparison to the appropriate isotype control as detailed on their websites. Briefly, Flk1-APC and VE-Cadherin-AF660, were tested for flow cytometry analysis on the bEnd-3 cell line. CD41-PE and CD41-FITC were tested by flow cytometry for staining on mouse platelets while CD19-PE and CD45-BV605 were tested using mouse splenocytes. CD44-PE and CD117-BV421 were tested on C57BL/6 mouse bone marrow cells and CD43-PerCP-cy5.5 was tested on the bone marrow cells of BALB/c mice. Furthermore, CD19-PE, CD4-PEcy7 and CD8a-FITC were tested upon mouse splenocytes prior to use in our lab. Additionally, fluorescence minus one (FMOs) were used in all flow cytometry experiments to control for background staining.

The blocking specificity of the KM201 CD44 antibody was previously demonstrated (Miyake, K. et al., 1990). Abcam validated the polyclonal CD44 antibody for immunofluorescence staining. The VE-Cadherin antibody staining has been tested in conjunction with a VE-Cadherin-GFP mouse line (Qiao et al., 2013).

Eukaryotic cell lines

Policy information about cell lines

Cell line source(s)

Mouse ESCs were derived from E3.5 blastocysts.

Authentication

Mouse ESCs line was authenticated according to its capacity of differentiating in mesoderm, endothelial and blood cells (see Figure 9).

Mycoplasma contamination

The line tested negative for mycoplasma.

Commonly misidentified lines
(See ICLAC register)

Name any commonly misidentified cell lines used in the study and provide a rationale for their use.

Animals and other organisms

Policy information about studies involving animals; ARRIVE guidelines recommended for reporting animal research

Laboratory animals

C57BL/6 male and female mice were used with age ranging from 5 to 12 weeks.

Wild animals

Provide details on animals observed in or captured in the field; report species, sex and age where possible. Describe how animals were caught and transported and what happened to captive animals after the study (if killed, explain why and describe method; if released, say where and when) OR state that the study did not involve wild animals.

Field-collected samples	*For laboratory work with field-collected samples, describe all relevant parameters such as housing, maintenance, temperature, photoperiod and end-of-experiment protocol OR state that the study did not involve samples collected from the field.*
Ethics oversight	*Identify the organization(s) that approved or provided guidance on the study protocol, OR state that no ethical approval or guidance was required and explain why not.*

Note that full information on the approval of the study protocol must also be provided in the manuscript.

Flow Cytometry

Plots

Confirm that:

- [x] The axis labels state the marker and fluorochrome used (e.g. CD4-FITC).
- [x] The axis scales are clearly visible. Include numbers along axes only for bottom left plot of group (a 'group' is an analysis of identical markers).
- [x] All plots are contour plots with outliers or pseudocolor plots.
- [x] A numerical value for number of cells or percentage (with statistics) is provided.

Methodology

Sample preparation	1. Cells derived from the AGM were dissected from mid-gestation mouse embryos. The tissue was digested for 30 minutes at 37 degrees Celsius in collagenase and then pipetted up and down to create a single cell suspension. Collagenase was deactivated with 10mL of "FACS buffer" consisting of 10% FBS in 1x PBS. Cells were centrifuged and washed before staining in conjugated antibodies.<br>2. Cells derived from the mouse ESC differentiation system were harvested by collecting the media and using TrypLE-express for 3 minutes to remove attached cells from the culture dish. The TrypLE was neutralised using an equal volume of FACS buffer before being centrifuged and washed. Cells were then stained with conjugated antibodies.
Instrument	Flow cytometry and sorting was performed on a BD FACSAria SORP (Beckton Dickinson, San Jose, CA)
Software	To collect and sort FACS data the FACS Diva Software (v 8.0.2).was used. Further analysis of the data was performed using FlowJo v10.4.2
Cell population abundance	Post-sort fractions were analysed using single cell qPCR and therefore purity of samples was determined by the expression levels of 96 genes.
Gating strategy	For all FACS experiments debris was removed by gating on size using SSC-A and FSC-A parameters. Then doublets were excluded from the samples using both FSC-H vs FSC-W and SSC-H vs SSC-W. Finally viability was determined by gating out cells that were positive for dead cells dyes Sytox Blue or 7-AAD. 7-AAD was also used to exclude auto-fluorescent erythrocytes. Further gating was determined based on FMO controls.

- [x] Tick this box to confirm that a figure exemplifying the gating strategy is provided in the Supplementary Information.
