# Supplementary material for: Single-cell transcriptomics identifies CD44 as a marker and regulator of endothelial to haematopoietic transition
Source: Nat Commun. 2020 Jan 29;11:586. doi: 10.1038/s41467-019-14171-5 (PMC6989687; doi:10.1038/s41467-019-14171-5)
Supplement: Supplementary file 4 — Description of Additional Supplementary Files [file 41467_2019_14171_MOESM4_ESM.pdf]

Description of Additional Supplementary Files

Supplementary Data 1: Expression Data from single-cell RNA-seq from Fig. 1d
The first worksheet contains log10(1+TPM) expression data from single-cell RNA-seq and the second the metadata relative to the cells shown in Fig. 1d.

Supplementary Data 2: Results of single-cell q-RT-PCR from Supplementary Fig. 4
The first worksheet contains log2 expression data from single-cell q-RT-PCR and the second the metadata relative to the cells shown in Supplementary Fig. 4.

Supplementary Data 3: Results of single-cell q-RT-PCR from Fig. 3 and Supplementary Fig. 5
The first worksheet contains log2 expression data from single-cell q-RT-PCR and the second the metadata relative to the cells shown in Fig. 3 and Supplementary Fig. 5.

Supplementary Data 4: Results of single-cell q-RT-PCR from Fig. 4 and Supplementary Fig. 7
The first worksheet contains log2 expression data from single-cell q-RT-PCR and the second the metadata relative to the cells shown in Fig. 4 and Supplementary Fig. 7.

Supplementary Data 5: Results of the RNA sequencing from Fig. 5
First worksheet: Matrix showing rlog transformed expression values after normalization with the DSEQ2 package.
Second worksheet: Metadata related to the samples in Fig.5
Third worksheet: Gene list resulting from the differential expression analysis between the CD44Neg and CD44LowKitNeg populations (p-value_adjusted <0.01). The results were obtained following the Wald statistical test. Negative LogFC values indicate higher gene expression in CD44LowKitNeg compared to CD44Neg while positive LogFC values indicate higher expression in CD44Neg compared to CD44LowKitNeg.
Fourth worksheet: Expression matrix used in Fig. 5b.
Fifth worksheet: Expression matrix used in Fig. 5c.
Sixth worksheet: Expression matrix used in Fig. 5d.

Supplementary Data 6: Single-cell RNA-seq Expression Data from Supplementary Fig. 8
The first worksheet contains log10(1+TPM) expression data from single-cell RNA-seq and the second the metadata relative to the cells shown in Supplementary Fig. 8.

Supplementary Data 7: Single-cell RNA-seq Expression Data from Supplementary Fig. 9
The first worksheet contains log10(1+NormalisedCounts) expression data from single-cell RNA-seq and the second the metadata relative to the cells shown in Supplementary Fig. 9.

Supplementary Data 8: Results of the reporter metabolite analysis from Fig. 6 and Supplementary Fig. 13
the first worksheet contains a table listing the results of the reporter metabolite analysis generated from the comparison of differentially expression genes between CD44Neg and CD44LowKitNeg populations.

Supplementary Data 9: Results of single-cell q-RT-PCR from Supplementary Fig. 14
The first worksheet contains log2 expression data from single-cell q-RT-PCR and the second the metadata relative to the cells shown in Supplementary Fig. 14.
